# Use of machine learning to identify novel, behaviorally active antagonists of the insect odorant receptor co-receptor (Orco) subunit

**DOI:** 10.1038/s41598-019-40640-4

**Published:** 2019-03-11

**Authors:** Devin Kepchia, Pingxi Xu, Raymond Terryn, Ana Castro, Stephan C. Schürer, Walter S. Leal, Charles W. Luetje

**Affiliations:** 1 Department of Molecular and Cellular Pharmacology, University of Miami Miller School of Medicine, Miami, Florida 33136 USA; 2 Department of Molecular and Cellular Biology, University of California, Davis, CA 95616 USA; 3 Center for Computational Science, University of Miami, Coral Gables, FL 33146 USA

## Abstract

Olfaction is a key component of the multimodal approach used by mosquitoes to target and feed on humans, spreading various diseases. Current repellents have drawbacks, necessitating development of more effective agents. In addition to variable odorant specificity subunits, all insect odorant receptors (ORs) contain a conserved odorant receptor co-receptor (Orco) subunit which is an attractive target for repellent development. Orco directed antagonists allosterically inhibit odorant activation of ORs and we previously showed that an airborne Orco antagonist could inhibit insect olfactory behavior. Here, we identify novel, volatile Orco antagonists. We functionally screened 83 structurally diverse compounds against Orco from *Anopheles gambiae*. Results were used for training machine learning models to rank probable activity of a library of 1280 odorant molecules. Functional testing of a representative subset of predicted active compounds revealed enrichment for Orco antagonists, many structurally distinct from previously known Orco antagonists. Novel Orco antagonist 2-*tert*-butyl-6-methylphenol (BMP) inhibited odorant responses in electroantennogram and single sensillum recordings in adult *Drosophila melanogaster* and inhibited OR-mediated olfactory behavior in *D. melanogaster* larvae. Structure-activity analysis of BMP analogs identified compounds with improved potency. Our results provide a new approach to the discovery of behaviorally active Orco antagonists for eventual use as insect repellents/confusants.

## Introduction

Insect borne diseases, such as malaria, dengue and Zika, are major concerns for human health and wellbeing. The most effective and widely used insect repellent is *N*,*N*-diethyl-*m*-toluamide (DEET)^1,2^. However, the low potency of DEET requires use of high concentrations to achieve adequate repellency, which can lead to skin irritation in sensitive individuals. More importantly, DEET is too expensive for widespread use in endemic regions. In addition, mosquitoes can exhibit reduced repellency by DEET upon repeated exposure^3^. Other currently available repellents do not offer greater effectiveness^2^. Thus, there is a need for development of new repellents/confusants that can make a greater impact on the spread of insect borne diseases.

Disease vector mosquitoes use a variety of olfactory cues, as well as heat and visual cues, to locate and feed on humans^4^. Olfactory cues are detected using receptors from several families, including odorant receptors (ORs), glutamate receptor-like IRs and some members of the gustatory receptor (GR) family^5–11^. Effective control of disease vector mosquitoes will require a multimodal attack^10^, targeting multiple receptor families. Including the ORs in this attack is critical, as ORs help mediate preference for humans and final targeting to specific skin regions^6,8,10,12,13^. Targeting insect ORs with volatile inhibitors may allow manipulation of blood-feeding behavior.

Insect ORs are embedded in the plasma membranes of olfactory sensory neurons (OSNs) located in the antennae and maxillary palps^14^. These receptors function as ligand (odorant) gated, non-selective cation channels^15,16^. ORs are tetrameric complexes^17^ containing variable odorant specificity subunits and a conserved odorant receptor co-receptor (Orco) subunit, in an unknown stoichiometry^18–20^. While both subunit types contribute to channel pore properties^21–23^, the odorant specificity subunits are the major determinant of odorant sensitivity and specificity^6,11,24–26^. Orco may serve a modulatory role, as dephosphorylation of Orco is proposed as a mechanism of odorant induced OR desensitization^27,28^. Orco is an obligate component of all functional ORs and is highly conserved across insect species^29–33^, making it an attractive target for the development of new compounds that manipulate insect olfactory behavior.

A recently discovered ligand-binding site on Orco allows modulation of OR function^34–39^. This ligand-binding site was revealed by the discovery of a compound, N-(4-ethylphenyl)-2-((4-ethyl-5-(3-pyridinyl)-4H-1,2,4-triazol-3-yl)thio)acetamide (VUAA1), that activated ORs through the Orco subunit^37^. A potential binding site is apparent in the recently published structure of a tetrameric Orco homomer^17^. Structural requirements for Orco agonists appear to be stringent, with even minor modifications often abolishing agonist activity^34,38,40,41^. Combined with the large size and lack of volatility of known Orco agonists, this currently limits opportunities for development of these compounds as airborne insect repellents. However, a large number of Orco antagonists have been discovered, some of which possess volatility^34–36,38,39^. Importantly, many of these Orco antagonists have been shown to inhibit odorant activation of ORs via an allosteric mechanism. Due to the high homology of Orco across species^19,30–33^, sufficiently volatile Orco antagonists could function as broad-spectrum insect repellents/confusants. We recently demonstrated that an airborne Orco antagonist can indeed inhibit OR-mediated insect olfactory behavior^42^, indicating that the further exploration of Orco antagonist structures is warranted.

Computational, or virtual, screening^43^ is widely used in early stage drug discovery to select likely active compounds. Among the various virtual screening approaches, machine learning is an established technique^44^ with various methodologies having been applied successfully^45^. A well performing example is the application of frequentist Bayesian classifiers using topological fingerprints as molecular descriptors; an approach that is computationally efficient, easy to interpret and relatively robust to noise and overfitting^46^. Virtual screening has also been applied in the olfactory space^47^ and machine learning has predicted odorant-receptor interactions, as well as “repellency”, using various sets of descriptors^48–51^. However, machine learning based on topological descriptors has not been used to identify novel odorant ligands. Such methods could be complicated due to the large receptor space and consequent structural ligand diversity and their generally low affinity. Computational screening, and in particular machine learning, has not been previously applied to the discovery of novel Orco ligands. Here, a simple machine learning approach based on ligand topology is demonstrated to identify a series of novel Orco antagonists, two of which are shown to be behaviorally active in an airborne context.

## Results

### Assembly of a diverse set of Orco antagonists with a wide range of potencies

**Figure 1:**
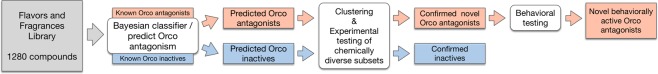
The strategy for identifying novel Orco antagonist structures with behavioral activity.

The strategy for identifying novel Orco antagonists was to develop a structurally diverse set of empirically determined active and inactive compounds for training machine learning classifiers to computationally rank 1280 compounds from the Sigma-Aldrich Flavors and Fragrances catalog. From these predictions, a test set of compounds would be chosen as structures which broadly represented the distribution of molecules with the highest and lowest predicted probability of being active (Fig. 1). A potential concern with this approach is that some of the compounds in our training set may also interact with odorant specificity subunits, activating or antagonizing some heteromeric ORs and potentially confounding our results. For example, S-carvone and 1-octanol have each been shown to activate or inhibit several different ORs from *D. melanogaster* and *A. gambiae*^6,11,24,52,53^. To avoid such complications, we chose to use homomeric channels formed by *A. gambiae* Orco (Agam\Orco), expressed in *Xenopus laevis* oocytes and assayed by two-electrode voltage clamp electrophysiology, as the experimental model for discovery of training compounds and for testing of predicted actives/inactives. Agam\Orco was activated by the Orco agonist 2-((4-Ethyl-5-(4-pyridinyl)-4H-1,2,4-triazole-3-yl)sulfanyl)-N-(4-isopropylphenyl)acetamide (OLC12)^34^. OLC12 is similar in structure to VUAA1^37^, but with a nitrogen in the 4 position (vs. the 3 position) of the pyridine ring and a 4-isopropyl moiety (vs. a 4-ethyl moiety) on the phenyl ring (Fig. S1).

**Figure 2:**
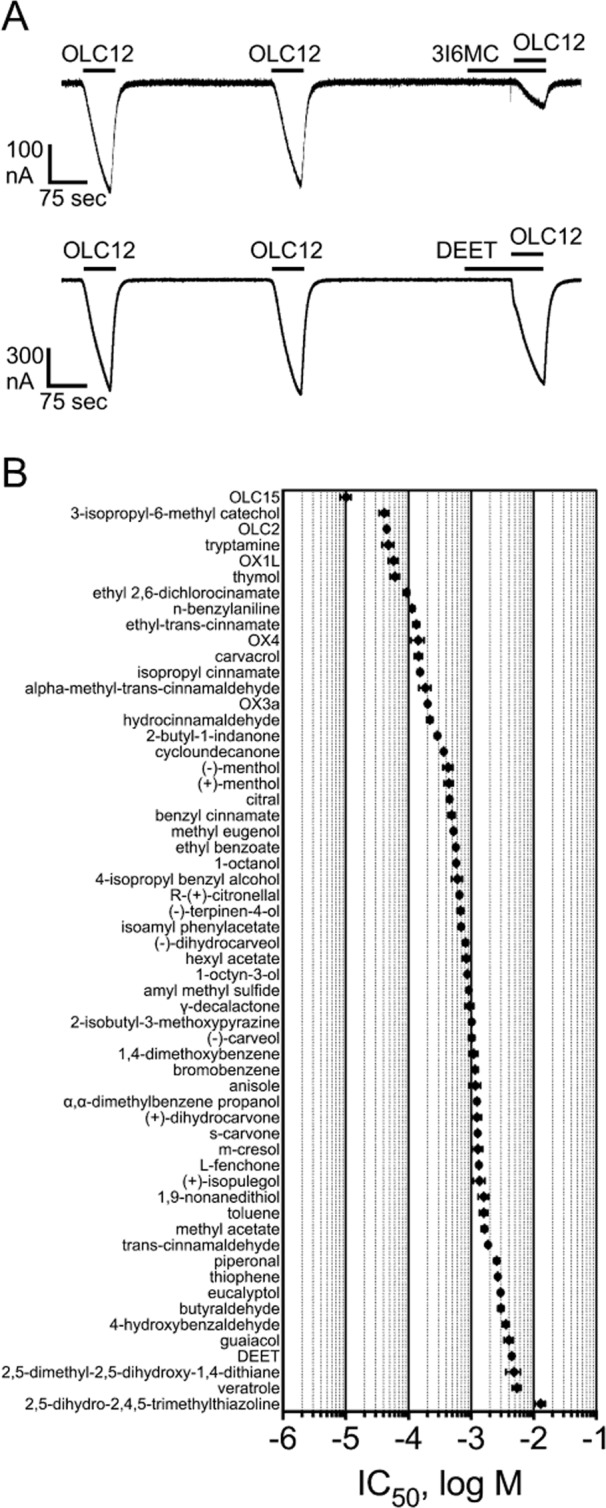
Structurally diverse compounds antagonize Orco with a range of potencies. (**A**) *Upper trace*, 3-isopropyl-6-methyl catechol (3I6MC) strongly inhibits OLC12 activation. An oocyte expressing Agam\Orco was challenged with two 60 sec applications of 30 µM OLC12, with 5 min wash periods between applications. 100 µM 3I6MC was then applied for 90 sec before a second application of OLC12 and co-applied during the OLC12 application. *Lower trace*, DEET causes little or no inhibition of OLC12 activation. An oocyte expressing Agam\Orco was challenged with two 60 sec applications of 30 µM OLC12. 1 mM DEET was then applied for 90 sec before a second application of OLC12 and co-applied during the OLC12 application. (**B**) IC_50_ values of compounds tested on *A. gambiae* Orco. Data are presented as mean ± SEM. (n = 3–8).

A structurally diverse panel of compounds was assembled and tested for antagonist activity at Agam\Orco. OLC12 activation of Agam\Orco in the presence of various concentrations of the antagonist candidates was compared to the response to OLC12 alone and the result expressed as a percentage. Figure 2A shows example traces for a highly effective antagonist 3-isopropyl-6-methyl catechol (3I6MC) which strongly inhibited OLC12 activation when applied at a concentration of 100 µM, as well as an ineffective compound *N,N*-diethyl-*m*-toluamide (DEET), which caused little or no inhibition when applied at a concentration of 1 mM. The lack of responses to antagonist alone, even at high concentrations, allowed us to ensure that antagonist candidates were not causing non-specific effects on the oocytes that might confound our results (see examples during application of antagonist alone to Agam\Orco expressing oocytes prior to co-application with agonist in Fig. 2A and application to sham (water) injected oocytes in Fig. S2).

Our screening panel was composed of compounds previously shown to antagonize Orco from other insect species^34–36^, known insect repellents ((R)-(+)-citronellal and DEET), and a series of compounds chosen based on a recent report that cinnamate-based structures (such as ethyl-*trans*-cinnamate and isopropyl cinnamate) and carvacrol function as Orco antagonists^39^. Of particular interest were known odorants and related structures with volatilities suitable for eventual behavioral testing in an airborne context. Of the candidate Orco antagonist molecules tested on Agam\Orco, 3I6MC was one of the most potent, with an IC_50_ of 41 ± 7 µM. Removing the hydroxyl adjacent to the methyl (thymol) slightly decreased potency, while instead removing the hydroxyl adjacent to the isopropyl (carvacrol) resulted in a greater decrease in potency (3.6-fold). Structures with a saturated ring (menthols) were 7-fold less potent than the structure with a benzene ring (thymol). Additional related structures were also tested, yielding a range of potencies. Cinnamate-based structures also displayed a range of potencies. The most potent compound was ethyl-*trans*-cinnamate, followed by isopropyl cinnamate, α-methyl*-*cinnamaldehyde, benzyl cinnamate, and *trans*-cinnamaldehyde. To further expand the panel of structures with Orco antagonist activity, as well as to identify compounds lacking Orco antagonist activity, a diverse panel of 46 compounds was also screened. Ultimately, 58 structurally diverse Orco antagonists, with potencies ranging from 10 ± 2 µM to 13 ± 2 mM, were identified as training actives (Fig. 2B, Table S1). For training inactives, 25 structurally diverse compounds that lacked antagonist activity on Agam\Orco (defined as ≤20% inhibition at 3 mM) were also identified (Table S2).

A variety of Orco antagonists have been shown to exert allosteric inhibition of odorant activation of a wide range of insect ORs^34–36,38,42^. Similarly, we found that 3I6MC could inhibit odorant activation of three different ORs from *A. gambiae* (Agam\Or28 + Agam\Orco, Agam\Or39 + Agam\Orco, Agam\Or65 + Agam\Orco), an OR from *Culex quinquefasciatus*, the southern house mosquito (Cqui\Or21 + Cqui\Orco) and an OR from *Drosophila melanogaster* (Dmel\Or35a + Dmel\Orco), when each OR was activated by its cognate odorant agonist (Fig. S3). We also found that 3I6MC could antagonize OLC12 (Orco agonist) activation of heteromeric ORs from *C. quinquefasciatus* and *D. melanogaster* (Fig. S3).

### Use of machine learning to prioritize potential Orco antagonists for functional testing

**Figure 3:**
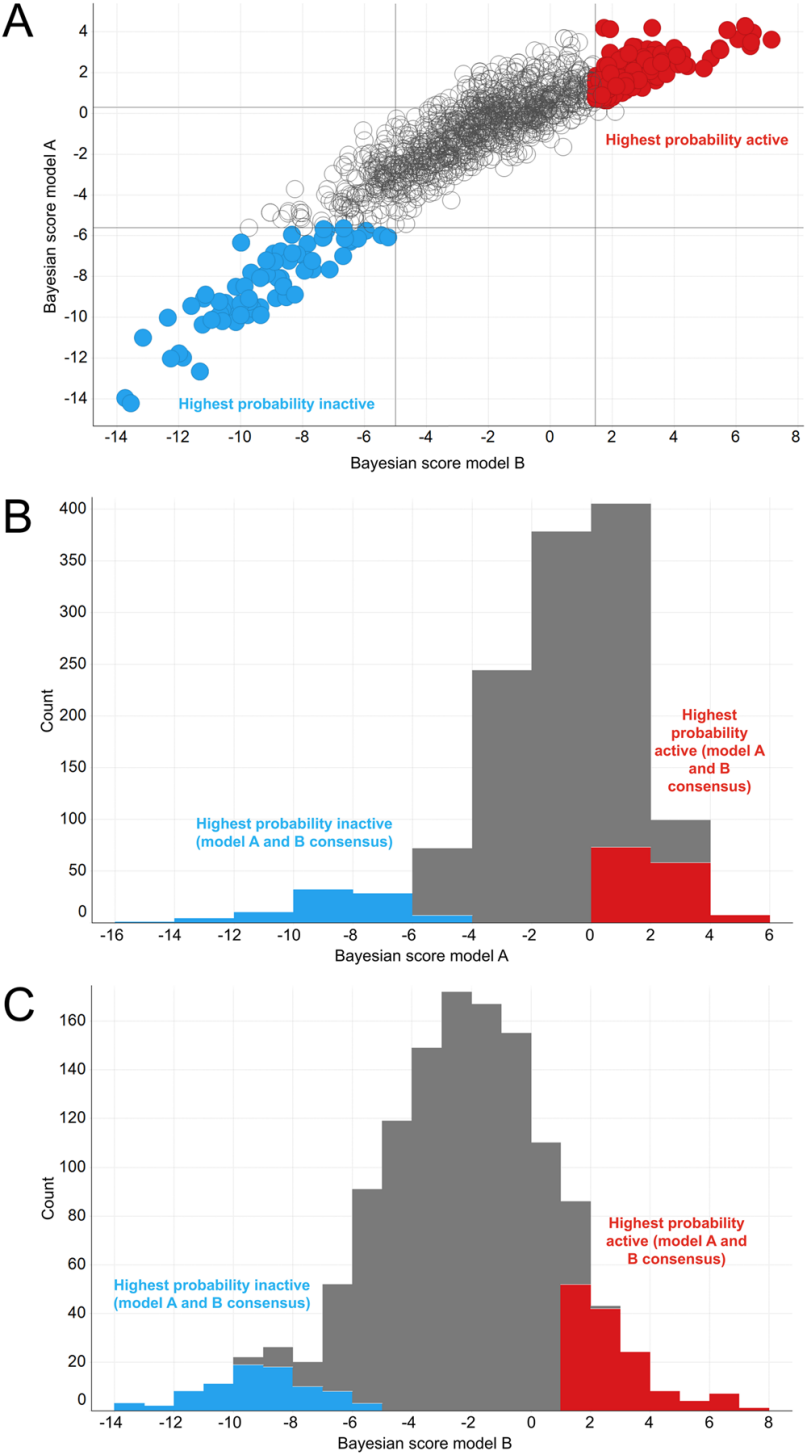
(**A**) Scatter plot of predictions (Bayesian scores) of model A (all actives vs inactives) and model B (most potent actives vs inactives) for 1280 compounds from the Sigma Aldrich Flavor and Fragrance catalog. The highlighted 138 highest probability actives (red) were selected by EstPGood predictions >0.9 for both models. The highlighted 82 highest probability inactives (blue) were selected by EstPGood <0.1 for both models. (**B**) Histogram of predictions (Bayesian scores) of model A, showing all predicted actives (red) and predicted inactives (blue) from panel A. (**C**) Histogram of predictions (Bayesian scores) of model B, showing all predicted actives (red) and predicted inactives (blue) from panel A.

To more effectively identify novel Orco antagonists, and to prioritize novel ligands for functional testing, machine learning classifiers were developed by using Orco antagonist activity data. Using standardized structures of the panel of 83 compounds (58 active antagonists and 25 inactives), two different classifiers were constructed to accommodate the wide range of antagonist potencies. The first classifier (A) was trained using all 58 actives and all 25 inactives. For the second classifier (B), only antagonists with IC_50_ values lower than 500 μM (21 compounds) were used as actives, while all 25 inactives were used. Laplacian modified Naïve Bayesian classifiers were used in combination with Extended Connectivity Fingerprints (ECFP4)^54^. Our models (A and B) had an estimated receiver operating characteristic (ROC) area under the curve (AUC) of 0.89 and 0.95, respectively, based on leave one out cross validation of the Pipeline Pilot learner (Table S3). Randomizing the labels of the datasets resulted in average ROC scores of 0.57 and 0.58 respectively (100 repetitions), further validating the models. We also performed k-fold stratified cross validations that yielded ROC scores in line with the scores estimated by leave one out validation (0.96 to 1.0, Table S3). Upon validation, the models were used to rank-order 1280 compounds from the Sigma-Aldrich Flavors and Fragrances catalog (Fig. 3).

### Structurally novel Orco antagonists predicted using machine learning models

**Figure 4:**
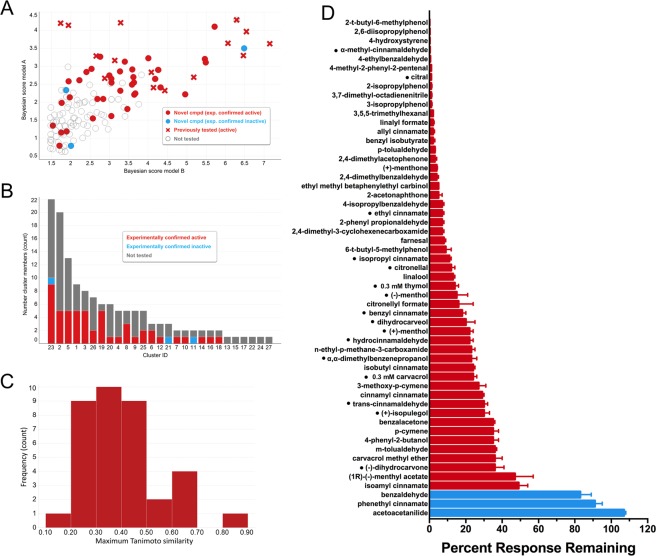
(**A**) Scatter plot of predictions (Bayesian scores) from model A and model B for the 138 highest probability predicted actives. Red solid circles, experimentally confirmed as active; blue solid circles, experimentally shown to be inactive; grey circles, not tested; solid circles represent novel compounds (not previously tested for Orco antagonist activity); solid crosses represent previously tested compounds (part of the training set). (**B**) Cluster membership of the 138 most likely predicted actives and distribution of experimentally tested compounds. (**C**) Histogram of maximum pairwise similarity of 36 novel Orco antagonists to the previously known actives. For each of the 36 novel Orco antagonists, the most similar previously known active was identified and the maximum Tanimoto similarity determined. (**D**) Antagonist activity of 39 predicted Orco antagonists, as well as the activity of 16 known Orco antagonists (denoted by a dot (•) preceding the compound name). The current response to 30 µM OLC12 application in the presence of 3 mM compound was compared to the average of two preceding responses to OLC12 alone and is presented as a percentage, after normalization to the results of a sham application. Thymol and carvacrol were tested at 300 µM, due to oocyte toxicity at higher concentrations. Compounds able to reduce the OLC12 response by more than 20% were classified as active (red bars). Compounds that reduced the OLC12 response by 20% or less were classified as inactive (blue bars). Data are presented as mean ± SEM (n = 3–10).

To identify novel, structurally diverse compounds using the machine learning classifiers, the most likely actives were first selected based on the (predicted) estimated probability of compounds being active (EstPGood) for both models. These most likely predicted actives were then clustered by structural similarity and representative compounds were selected from each cluster for testing. Specifically, a value of EstPGood >0.9 was used as a cutoff for both models resulting in 138 pre-filtered structures which were grouped into 27 clusters by selecting an average number of 5 members per cluster. The 138 highest scoring compounds included 16 known actives that were part of the training set. Given that information, 39 additional (new) compounds were manually selected such that at least one compound from each cluster (omitting singletons) was represented among the most likely predicted actives. In addition to structural diversity (by clustering), our selection criteria included molecular weight, the predicted probabilities, cluster size, and availability (Fig. 4A,B). The vast majority of the 39 compounds (36) were found to be active, with only three inactives, suggesting that the machine learning classifiers were highly predictive. Specifically, 25 of the 36 novel actives elicited 80% or greater inhibition of Orco activity, and all 36 inhibited the Orco response by more than 50% (Fig. 4D).

The structural novelty of the novel confirmed active Orco antagonists was assessed by maximum pairwise Tanimoto similarity (Fig. 4C). When compared to the previously known Orco antagonists, only seven of the 36 novel antagonists had a maximum similarity of greater than 0.5 (one was >0.8, four were 0.6–0.8), whereas the other 29 compounds had a maximum similarity of less than 0.5 (20 less than 0.4). These results show that our models were able to identify structurally novel Orco antagonists.

**Figure 5:**
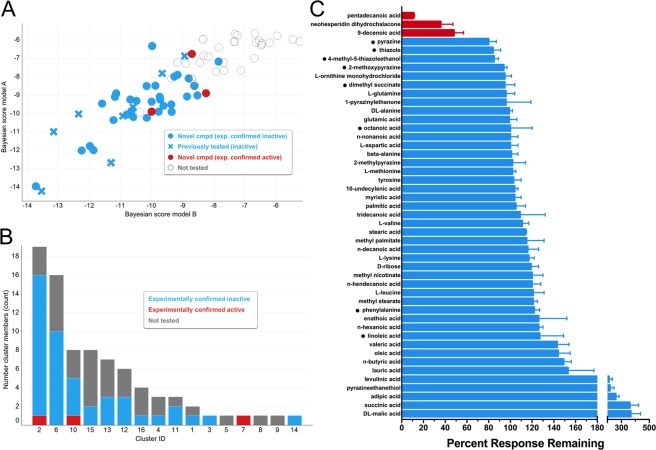
(**A**) Scatter plot of predictions (Bayesian scores) from model A and model B for 82 highest probability inactives. Blue solid circles, experimentally confirmed as inactive; red solid circles, experimentally shown to be active; grey circles, not tested; solid circles represent novel compounds (not previously tested for Orco antagonist activity); solid crosses represent previously tested compounds (part of the training set). (**B**) Cluster membership of the 82 most likely predicted inactives and distribution of experimentally tested compounds. (**C**) Antagonist activity of 39 compounds predicted to lack Orco antagonist activity, as well as the activity of 8 compounds known to lack Orco antagonist activity (denoted by a dot (•) preceding the compound name). The current response to 30 µM OLC12 application in the presence of 3 mM compound was compared to the average of two preceding responses to OLC12 alone and is presented as a percentage, after normalization to the results of a sham application. Compounds that reduced the OLC12 response by 20% or less were classified as inactive (blue bars). Compounds able to reduce the OLC12 response by more than 20% were classified as active (red bars). Data are presented as mean ± SEM (n = 3–7).

As a negative control, a diverse subset of most likely inactive compounds was also selected by first filtering for EstPGood <0.1 for both models and clustering the resulting 82 compounds (which included eight previously tested inactives that were part of the training set) into 16 clusters with an average of five members per cluster. 39 additional (new) compounds, selected to represent all clusters except singletons (Fig. 5A,B), were then functionally tested in the Orco antagonism assay (Fig. 5C). Of the 39 compounds, 36 were found to be inactive and three active. This result further indicated that the machine learning classifiers were highly predictive.

It is noteworthy that the Laplacian-corrected Naïve Bayesian machine learning approach in combination with extended connectivity fingerprints correctly predicted structurally novel small molecule Orco antagonists and – as control – also identified inactives. We defined chemical structural novelty based on Tanimoto similarity using the same descriptors that underlie the classifiers. These results illustrate the utility of the circular topological fingerprints as descriptors for small molecules in this chemical space and the effectiveness of the relatively simple frequentist Naïve Bayesian method in weighting the structural features to differentiate actives and inactives with high accuracy.

In the next step, ten of the novel Orco antagonists showing >80% inhibition at 3 mM were assessed for potency (IC_50_) using concentration-inhibition analysis (Table 1). One aromatic and one non-cyclic aliphatic compound were selected for further study in an *in vivo* context: 2-*tert*-butyl-6-methylphenol (BMP), with an IC_50_ of 48 ± 5 μM; and linalyl formate (LF), with an IC_50_ of 251 ± 39 μM. Neither BMP or LF exerted any non-specific effects when applied at 3 mM to sham (water) injected oocytes (Fig. S2). Similar to what we showed above for 3I6MC (Fig. S3) and to what has been previously shown for a variety of Orco antagonists^34–36,38^, BMP and LF were able to inhibit multiple ORs from various species. Both BMP and LF were able to inhibit odorant activation of three different ORs from *A. gambiae* (Agam\Or28 + Agam\Orco, Agam\Or39 + Agam\Orco, Agam\Or65 + Agam\Orco), an OR from *C. quinquefasciatus* (Cqui\Or21 + Cqui\Orco) and an OR from *D. melanogaster* (Dmel\Or35a + Dmel\Orco), when each OR was activated by its cognate odorant agonist (Fig. S3). Both BMP and LF were also able to antagonize OLC12 (Orco agonist) activation of heteromeric ORs from *C. quinquefasciatus* and *D. melanogaster* (Fig. S3). This functional conservation of Orco across species^22,34–36,42^ allowed us to test compounds we identified with Orco from *A. gambiae* using well-established *in vivo* assays in *D. melanogaster*.

**Table 1:** Ten newly identified compounds that displayed high Orco antagonist activity (greater than 80% inhibition at 3 mM) in Fig. 4D were further investigated by concentration-inhibition analysis.

Compound	IC_50_ (μM)	Estimated Vapor Pressure (mmHg at 25 °C)	Boiling Point (degrees C)
	47 ± 3	0.008	261.4
	48 ± 5	0.03	247.7
	135 ± 15	0.09	218.3
	195 ± 18	0.04	244.7
	251 ± 39	0.2	211.7
	262 ± 24	0.04	209.2
	262 ± 29	0.01	264.3
	527 ± 84	0.29	201.5
	554 ± 51	0.13	220.9
	719 ± 48	0.08	204.1

IC_50_ values for inhibition of Agam\Orco are presented as mean ± SEM (n = 3–8). Vapor pressures and boiling points were estimated as described in Methods.

### Inhibition of *in vivo* electrophysiological responses by a novel Orco antagonist

**Figure 6:**
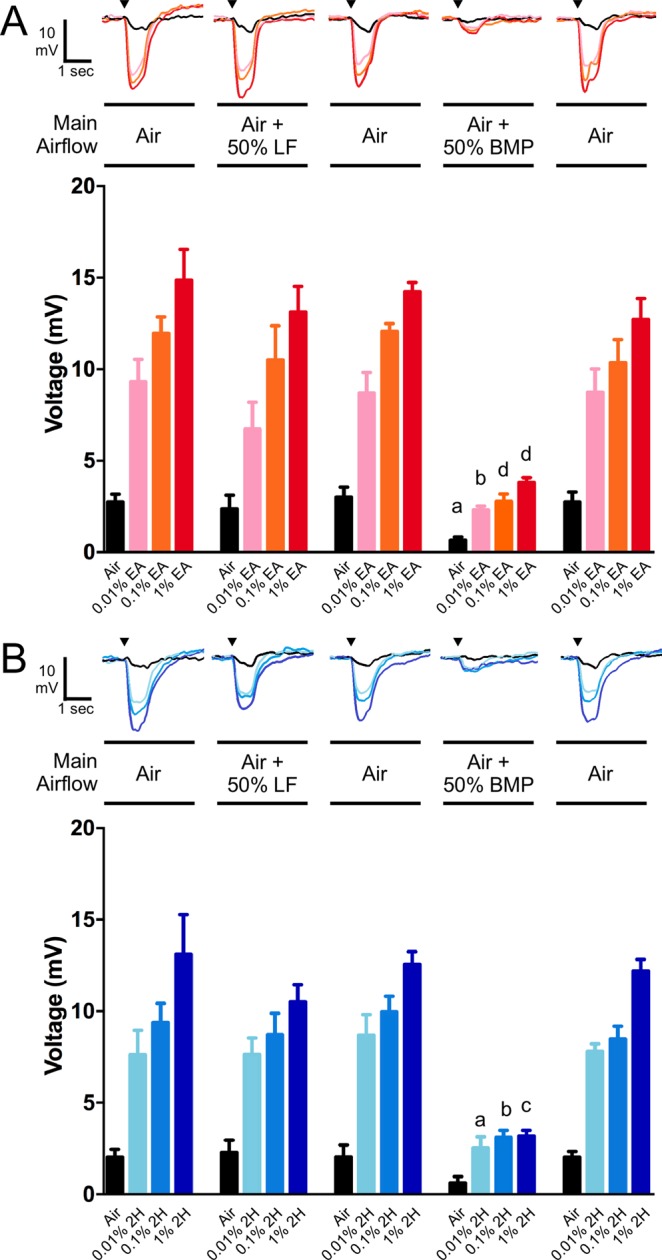
Inhibition of odorant induced EAG responses of fruit fly antennae by an Orco antagonist. (**A**) *Top*, EAG recordings from a single insect exposed sequentially to stimulus pulses of air, 0.01% EA, 0.1% EA and 1% EA. The stimulus pulse set was repeated with the main airflow containing air, air + 50% LF, air, air + 50% BMP, air. *Bottom*, graphic representation of repetitions (n = 3–5) of the experiments shown above. Data are presented as mean ± SEM. (**B**) *Top*, EAG recordings from a single insect exposed sequentially to stimulus pulses of air, 0.01% 2 H, 0.1% 2 H and 1% 2 H. The stimulus pulse set was repeated with the main airflow containing air, air + 50% LF, air, air + 50% BMP, air. *Bottom*, graphic representation of repetitions (n = 3) of the experiments shown above. Data are presented as mean ± SEM. Statistical analysis: The stimulus pulses of each type (air, 0.01% EA, etc.) were compared by one-way ANOVA and Dunnett’s multiple comparison test. For comparison to the stimulus pulse in the first main airflow set (Air): a, p < 0.05; b, p < 0.01; c, p < 0.001; d, <0.0001.

Electroantennogram (EAG) recordings in adult *D. melanogaster* flies were used to determine whether the novel Orco antagonists BMP and LF could inhibit olfactory responses in an *in vivo* context (Fig. 6). Stimulus pulse applications (see Methods) of the odorant agonists ethyl acetate (EA) and 2-heptanone (2 H) elicited antennal responses. Inclusion of LF (filter paper containing 10 µL of 50% LF in mineral oil) in the main airflow did not alter the amplitude of EAG responses to EA or 2 H. In contrast, inclusion of BMP (filter paper containing 10 µL of 50% BMP in mineral oil) in the main airflow significantly reduced the amplitude of EAG responses to EA and 2 H. BMP also significantly reduced the baseline response to ambient air (see Methods). When the EA or 2 H responses in the presence of BMP were represented as a percentage of the preceding response to EA or 2 H alone, the extent of inhibition was the same for the three different odorant concentrations (Fig. S4), suggesting a non-competitive mechanism for inhibition of these odorant responses. This is consistent with previous studies demonstrating that many different Orco antagonists inhibit odorant responses via an allosteric and non-competitive mechanism^34–36,38^. Furthermore, when we performed concentration-inhibition analysis of BMP in *X. laevis* oocytes, we found BMP to be a non-competitive antagonist of odorant activation of a heteromeric OR (Fig. S5).

**Figure 7:**
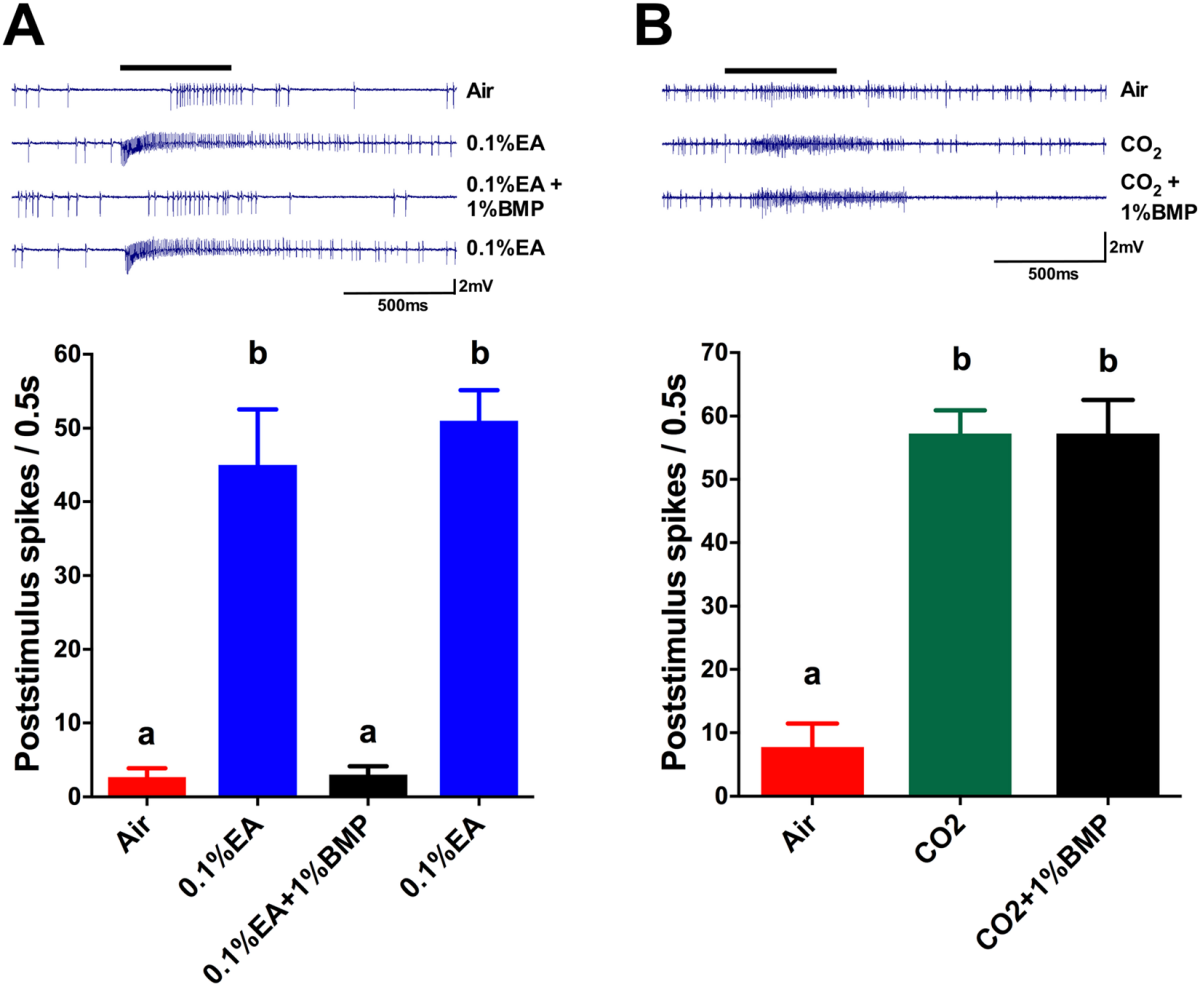
Inhibition of odorant induced, but not CO_2_ induced, SSR responses of fruit fly antennae by an Orco antagonist. (**A**) *Top*, SSR recordings from an ab2 sensillum of a single insect exposed sequentially to stimulus pulses (500 ms) of air, 0.1% EA, 0.1% EA + 1% BMP, and 0.1% EA. *Bottom*, graphic representation of repetitions (n = 3) of the experiments shown above. Data are presented as mean ± SEM and were compared by one-way ANOVA and Tukey’s multiple comparison test. 0.1% EA bars differ from Air (p < 0.001). Bars labeled with the same letters are not significantly different (p > 0.05). (**B**) *Top*, SSR recordings from an ab1 sensillum of a single insect exposed sequentially to stimulus pulses (500 ms) of air, CO_2_ (see Methods), and CO_2_ + 1% BMP. *Bottom*, graphic representation of repetitions (n = 3) of the experiments shown above. Data are presented as mean ± SEM and were compared by one-way ANOVA and Tukey’s multiple comparison test. CO_2_ and CO_2_ + 1% BMP bars differ from Air (p < 0.0001). Bars labeled with the same letters are not significantly different (p > 0.05).

A further concern about the action of BMP in an *in vivo* context is that the structure of BMP is somewhat similar to that of the general anesthetic propofol (2,6-diisopropylphenol), which also has Orco antagonist activity (Table 1). To determine whether the actions of BMP in an *in vivo* electrophysiological context were Orco dependent, we turned to single sensillum recording (SSR) in adult *D. melanogaster* flies (Fig. 7). The ab2 sensilla contains an EA responsive OSN^55^. When we recorded from the ab2 sensilla and applied EA (10 µL of 0.1% in mineral oil), a robust response was obtained. Inclusion of BMP in the airflow (10 µL of 1% in mineral oil) abolished the response (Fig. 7A). The effect of BMP was reversible, as the subsequent response to EA alone was fully restored. The ab1 sensilla contains an OSN that responds to CO_2_^55^, due to the expression of Gr21^56^ and Gr63a^57,58^. GRs are thought to be structurally related to ORs, but do not utilize Orco in receptor formation^57,58^. When we recorded from the ab1 sensilla and applied a CO_2_ stimulus (see Methods), a robust response was obtained. Inclusion of BMP in the airflow (10 µL of 1% in mineral oil) had no effect on the CO_2_ response (Fig. 7B). These results indicate that the inhibitory action of BMP in an *in vivo* electrophysiological context is via an Orco dependent mechanism.

### Inhibition of insect olfactory behavior by novel Orco antagonists

The robust attraction of *D. melanogaster* larvae to EA is OR dependent, as genetic deletion of Orco (preventing expression of all ORs), or of the Or42b odorant specificity subunit (which mediates high sensitivity to EA), completely abolishes this attraction^42^. We previously demonstrated that this OR-dependent olfactory behavior can be inhibited by an airborne Orco antagonist^42^. We now asked whether the two newly identified, structurally novel Orco antagonists (BMP and LF) were also able to alter this insect olfactory behavior.

**Figure 8:**
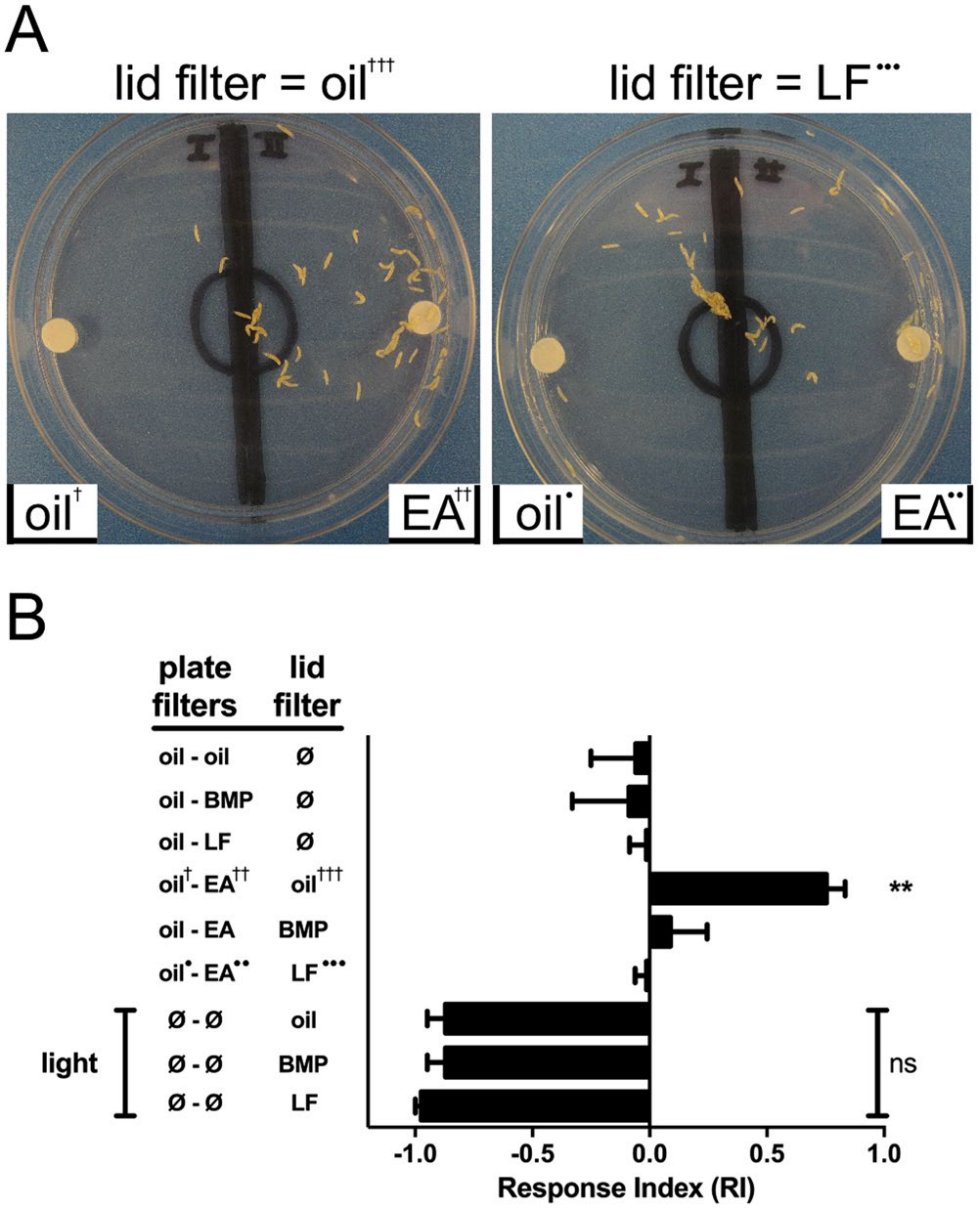
Orco antagonist molecules from two distinct structural groups can inhibit ethyl acetate attraction. (**A**) A representative linalyl formate inhibition experiment. In both panels, larvae were placed in the starting circle, flanked on the right by EA (10 µL of EA, diluted 1:10^−6^ in mineral oil) and on the left by mineral oil (10 µL). In the left panel, mineral oil (vehicle) was applied to the lid filter paper, while in the right panel, linalyl formate was applied to the lid filter paper. (**B**) Results of the larval chemotaxis assay. EA, ethyl acetate; oil, mineral oil (vehicle); BMP, Orco antagonist (2-*tert*-butyl-6-methylphenol); LF, Orco antagonist (linalyl formate); Ø, nothing added; light, fiber optic light source. Data are presented as mean ± SEM (n = 4–7). Results (top 6 bars) were compared by one-way ANOVA, followed by Dunnett’s multiple comparison test, for comparison to oil vs. oil control (top bar). When larvae were offered the choice of EA or mineral oil alone on the plate filters, the attraction to EA was strong, yielding a response index (RI) of 0.76 ± 0.08 (4^th^ bar from top), which significantly differed from the RI value obtained when mineral oil alone was offered on both plate filters (**p < 0.01). Application of BMP or LF to the lid filter (685 µL of 100 mM) completely abolished larval attraction to EA, yielding RI values that did not significantly differ from the RI value obtained when mineral oil alone was offered on both small filters. Light repulsion (bottom 3 bars) with mineral oil, BMP, or LF in the lid filter was compared by one-way ANOVA and Dunnett’s multiple comparison test (ns, no significant differences). Other symbols (†, •) are intended to allow the results in panel A to be placed within the context of panel B.

When larvae were offered the choice of EA (10 µL of EA, diluted 1:10^−6^ in mineral oil) or mineral oil alone, the attraction to EA was strong, yielding a response index (RI) of 0.76 ± 0.08 (Fig. 8A left panel, Fig. 8B), which significantly differed from the RI value obtained when mineral oil alone was offered on both filters. To ensure that BMP and LF would not elicit a behavioral response when applied alone, larvae were offered the choice of either BMP or LF (10 µL of 100 mM) vs. mineral oil vehicle. The larvae displayed no preference, with RI values that did not significantly differ from the RI value obtained when mineral oil alone was offered on both filters, indicating that BMP and LF are neither attractive or repulsive in this context.

To determine whether BMP or LF could affect the attraction of larvae to EA, 685 µL of 100 mM BMP or LF was added to a large filter paper adhered to the inner lid of the dish. In this configuration, BMP and LF must cross a 1-cm airspace in order to reach the larvae. We previously determined that 685 µL was the maximum volume that could be applied to the lid filter paper without concern about solution dripping onto the bottom of the plate^42^. Both BMP and LF completely abolished larval attraction to EA, yielding RI values that did not significantly differ from the RI value obtained when mineral oil alone was offered on both small filters.

When BMP and LF were each applied to the lid filter at the lower concentration of 30 mM, LF was again able to significantly reduce larval attraction to EA, while BMP did not significantly affect attraction to EA (Fig. S6). This result shows LF to be somewhat more potent than BMP in the behavioral assay. This contrasts with the results obtained in the functional assay with Agam\Orco (Table 1), where BMP was 5-fold more potent than LF, and the EAG assay (Fig. 6) where BMP, but not LF, inhibited odorant responses.

To ensure that BMP and LF were not exerting a generalized effect on the larvae, such as making them “sick” or disoriented, a different sensory modality was also tested, the *D. melanogaster* larval aversion to light^42,59^. When 685 µL of mineral oil vehicle was placed on the lid filter, the larvae were strongly repelled by a light source, resulting in a highly negative RI value. This aversion to light was unaffected when 685 µL of 100 mM BMP or 685 µL of 100 mM LF was placed on the lid filter, indicating that BMP and LF were not exerting a generalized effect on the larvae, but rather were specifically affecting olfactory behavior through an OR-dependent mechanism, consistent with their Orco antagonist properties.

### Structure-activity analysis of substituted phenol Orco antagonists

The bi-substituted phenol BMP, which was active as an inhibitor of odorant induced EAG and SSR responses (Figs 6 and 7) and as an inhibitor of olfactory behavior (Fig. 8), was one of the most potent of the novel Orco antagonists that we identified (Table 1). To explore this structural class in more detail, we performed concentration-inhibition analysis with nine additional mono-, bi- and tri-substituted phenols (Table 2). When we examined compounds with a 2,4-bi-substituted configuration, we found compounds with increases in potency. 2,4-di-*tert*-butyl-phenol was 3-fold more potent and 2-*tert*-butyl-4-methylphenol was 2-fold more potent than BMP. For 2,6-bi-substituted phenols, alterations in the 2- and/or 6-substitutions had either no effect on potency (2-*tert*-butyl-6-isopropylphenol) or slightly decreased potency (2,6-diethylphenol and 2,6-di-*tert*-butylphenol). Among the mono-substituted phenols, 2-*tert*-butylphenol showed a slight increase in potency, while 2-ethylphenol showed a 5.5-fold loss of potency. The only tri-substituted phenol tested (2,4,6-trimethylphenol) displayed a 4.5-fold loss in potency.

**Table 2:** Structure-activity analysis of substituted phenol compounds.

Compound	IC_50_ (μM)	Estimated Vapor Pressure (mmHg at 25 °C)	Boiling Point (degrees C)
	15 ± 1	0.003	281.2
	25 ± 3	0.01	247.7
	32 ± 5	0.05	229.7
	37 ± 7	0.003	261.4
	50 ± 7	0.001	271.4
	86 ± 17	0.008	247.7
	92 ± 9	0.007	281.2
	212 ± 29	0.02	229.6
	261 ± 14	0.15	210.7

IC_50_ values for inhibition of Agam\Orco are presented as mean ± SEM (n = 3–9). Vapor pressures and boiling points were estimated as described in Methods.

## Discussion

Insect ORs (and probably the GRs) constitute a novel class of ligand gated ion channel^15,17,18^. Due to the lack of a relationship to the receptors and channels of humans, insect ORs and Orco are attractive targets for new repellents/confusants with potentially low toxicity for humans. The divergence of odorant specificity subunit families allows each species to survey ecologically relevant portions of odor space to guide species-specific behavioral decisions^6^ and suggests odorant specificity subunits as targets for species-specific repellents/confusants^14,60^. However, the presence of the highly conserved Orco in all insect ORs suggests Orco as an attractive target for species-common repellents/confusants.

Orco is highly conserved across insect species and the Orco subunits from various species appear to be functionally interchangeable^22^. The ligand specificities of Orco subunits from many species are remarkably similar, suggesting a conserved ligand binding site^34–36^. The presence of Orco in all ORs and the ability of Orco antagonists to allosterically inhibit activation of ORs by odorants, suggests that Orco antagonists can serve as inhibitors of OR mediated olfaction in a broad range of insects. Whether the ligand binding site on Orco has a physiological role is not currently known, but the site may allow modulation of OR function by endogenous ligands^36^. If this modulatory role were critical, it would serve to limit the development of resistance to Orco ligands developed as repellents/confusants. Of course, broadly active Orco antagonists capable of inhibiting olfaction in many insects could be a cause for concern. Such compounds would affect deleterious insects (such as mosquitoes and agricultural pests), as well as beneficial insects (such as honeybees and other pollinators). Thus, broadly active Orco antagonists would be unsuitable for agricultural use. However, it is unlikely that an Orco antagonist developed for preventing mosquito bites would interfere with the ecology of beneficial insects, such as the honeybee. Beneficial insects are not attracted to humans and typically do not come in contact with humans, unless in self-defense. Only insects approaching the treated source (skin, clothing, homes, etc.) would be affected. Use of Orco antagonists in this manner would allow action at short-range on only those insects pursuing human contact, while avoiding negative effects on beneficial insect species. Furthermore, the Orco subunit of the Hessian fly (*Mayetiola destructor*) was recently found to be insensitive to the Orco agonist VUAA1^61^, suggesting that development of Orco ligands that only target deleterious insect species might be possible.

A large number of Orco antagonists have recently been identified^34–36,38,39^, but many of these compounds lack volatility. To be effective as an insect repellent/confusant, a compound should possess some volatility so that it can act in the air phase. However, highly volatile odorants would be unsuitable due to rapid evaporation and dissipation^2^. We recently demonstrated that an airborne Orco antagonist can inhibit olfaction-guided insect behavior^42^, but the thiophene constituent of this compound makes further development problematic, as thiophenes can be toxic. It was for these reasons that we sought to identify new Orco antagonists with novel structures and appropriate levels of volatility. We adopted a virtual screening approach using machine learning classifiers. This method is common in drug discovery^44,45^ and has been used to identify new odorants in insect olfaction^48,49,51^. This approach has not been previously applied to the discovery of Orco ligands. We found that Laplacian-corrected Naïve Bayesian machine learning classifiers developed using topological descriptors were highly predictive in enriching novel actives and inactives from a library of diverse odorant compounds (the Sigma-Aldrich Flavors and Fragrances catalog). Descriptors of small molecules for the purpose of similarity comparison and virtual screening via machine learning have been reviewed extensively in the drug discovery space^62–64^. Extended connectivity fingerprints are circular topological fingerprints and are considered of high utility in virtual screening and have been shown to work well with the Laplacien-corrected Naïve Bayesian classifiers. For that reason, ECFP4 fingerprints were our first choice of descriptor, along with basic physicochemical properties. This approach performed extraordinarily well in our study, identifying multiple structurally novel Orco antagonists. We purposefully did not consider pre-defined keys, because these are optimized for typical drug discovery projects and may not be as applicable to the Orco ligand chemical space, which is different from the drug space in terms of physicochemical properties and chemotypes. In addition, ECFP4 fingerprints are much more descriptive. However, we would expect that other descriptive topological fingerprints, such as path-based fingerprints will also perform well. Another general consideration would be pharmacophoric descriptors, which can be generated from 3D or 2D representations. Pharmacophoric descriptors can be applied to identify new chemotypes and are overall less descriptive; but could be an attractive option in the Orco ligand space. A further potentially attractive option in the Orco ligand space is shape-based virtual screening. Each of these approaches may merit future pursuit.

Our identification of a large number of new Orco antagonists that were structurally novel suggests that the machine learning approach will be generally useful in the further development of Orco ligands, and may also be useful in identifying ligands for odorant specificity subunits. Recent publication of a structure of an Orco homo-tetramer^17^ will allow the addition of virtual ligand docking techniques^65^ to the Orco ligand discovery effort.

The 45 novel Orco antagonist compounds shown in Fig. 4D and Table 2 have not been previously tested for Orco activity. However, because the target of our computational screening was the Sigma-Aldrich Flavors and Fragrances catalog, it is to be expected that some of the identified compounds might have been previously tested for activity as odorant ligands in insects. Indeed, seven of the 45 novel Orco antagonists (as well as four of the 16 input compounds appearing in Fig. 4D) have been shown to activate or inhibit ORs from *D. melanogaster* and/or *A. gambiae* (Table S4). Each of these compounds was only active at a small subset of ORs, indicating that the observed effect was due to interaction with variable odorant specificity subunits and not the constant Orco subunit. However, direct injection of an Orco antagonist during single sensillum recording (SSR) assays in *A. gambiae* has been shown to reduce the spontaneous firing rate of OR expressing neurons, as well as to inhibit odorant mediated responses^38^. Why then, have the compounds we identified as Orco antagonists not been seen to exert a uniform inhibitory effect? The answer lies with potency and concentration. The Orco antagonist (VU0183254) shown to reduce the spontaneous firing rate of OR expressing neurons^38^ was directly injected into sensilla at a concentration of ≥100 µM. In the *Xenopus* oocyte electrophysiology assay, this compound is 10- to 100-fold more potent than the 11 Orco antagonists listed in Table S4. In the SSR assay used in the studies summarized in Table S4, compounds must first vaporize into the air from small volume sources of dilutions ranging from 10^−2^ to 10^−6^, then pass through sensillar pores and dissolve into the sensillar lymph, perhaps interacting with odorant binding proteins (OBPs), before coming in contact with the ORs. While it is essentially impossible to know the concentrations that these compounds reach at the molecular target (the ORs) in the assay, it is unlikely that a concentration sufficient for Orco blockade would be achieved. Thus, these low potency Orco antagonists (the 11 compounds in Table S4) would not be expected to exert Orco mediated effects in these earlier studies.

When testing BMP and LF in the EAG and behavior assays, we chose to use very high amounts (10 µL of a 50% dilution in the EAG assay, 685 µL of 100 mM, approximately 2%, in the behavior assay) in order to provide as much advantage as possible to these compounds. When compared to the IC_50_ values for BMP and LF inhibition of Agam\Orco in the functional assay (48 ± 5 µM and 251 ± 39 µM, respectively), these amounts of BMP and LF might seem excessive. However, concentrations used in these assays can’t be easily compared. In the functional assay, the BMP or LF solution is applied directly to the Agam\Orco expressing oocytes, allowing certainty about the concentrations of these compounds at the molecular target. In the EAG and behavioral assays, the more circuitous route taken by the compounds (described in the previous paragraph) drastically reduces the amount of BMP or LF likely to reach the molecular target (the ORs) in these assays. Differences among Orco antagonists in volatility, sensillar lymph solubility and OBP affinity also means that while *in vitro* screening is effective in identifying suitable candidates for *in vivo* screening, an exact correspondence between *in vitro* potency and *in vivo* potency shouldn’t be expected. Also, the effectiveness of BMP and LF differed among the *in vivo* assays. While, LF was somewhat more potent than BMP in the behavioral assay, only BMP was active in the EAG assay. The higher volatility of LF may have offered an advantage in the passive diffusion/static air environment of the behavioral assay, rather than in the propelled airstream environment of the EAG assay. In addition, differences in the lymph/OBP environments of the two assays (the behavioral assay was performed in larvae, while the EAG assay was performed in adults) may have differentially affected the relative effectiveness of BMP and LF.

BMP, like many of the novel Orco antagonist compounds we have identified, has not been previously tested for OR activity. We found that while BMP did not act as an odorant agonist in the EAG experiments, it was able to inhibit EAG responses to two different odorant agonists, EA and 2 H (Fig. 6). BMP also suppressed the baseline response to ambient air. The inhibition of odorant responses by BMP in the EAG experiments appeared to be non-competitive (Fig. S4), a mechanism we confirmed in *in vitro* electrophysiology experiments (Fig. S5). In SSR experiments, BMP inhibited an OR mediated odorant response, but not a GR mediated CO_2_ response (Fig. 7). Taken together, these results are consistent with BMP’s actions as an Orco antagonist and suggest that the effect of BMP in the behavioral assay (Fig. 8) is due to action as an Orco antagonist and not due to action at odorant specificity subunits or at receptors lacking the Orco subunit. Furthermore, the failure of BMP to alter light aversion behavior, and the finding that BMP is neither attractive or repulsive, suggests that BMP is not generating a confusing or unpleasant signal via activation of a larval specific odorant specificity subunit. Also, the failure of BMP to alter the GR mediated CO_2_ response in the SSR experiments or the light aversion response in the behavioral assay belies the concern that BMP might be exerting an anesthetic effect due to its structural similarity to propofol.

We have found that two of the newly identified and structurally distinct Orco antagonists, BMP and LF, were behaviorally active. These compounds, when applied in an airborne context, could abolish the OR-mediated attraction of *D. melanogaster* larvae to ethyl acetate. LF, with an estimated vapor pressure likely to be too high to be an effectively long-lasting repellent (0.2 mmHg), also failed to exert an effect in the EAG assay, suggesting that this structural class might not be worth pursuing. In contrast, BMP was active in the EAG, SSR and behavioral assays, and had a lower estimated vapor pressure (0.03 mmHg), suggesting that this or similar compounds might be suitably long acting. By functionally screening a series of structural analogs of this compound, we identified a 2,4-bi-substituted phenol (2,4-*di*-*tert*-butylphenol) as a highly potent Orco antagonist with an estimated vapor pressure similar to that of DEET (0.003 mmHg). Further investigation of this structural class of Orco antagonist is likely to be productive. As more potent and appropriately volatile Orco antagonists are discovered, an expansion of behavioral studies to include disease-vector mosquitoes will be essential.

## Methods

### Materials

The PubChem CID number and vendor source for each compound tested for Orco antagonist activity in this study is provided in Table S5. Agam\Orco in pT7TS was generously provided by L. Zwiebel. Vapor pressures and boiling points of antagonist compounds were estimated using the Estimation Program Interface provided by the Environmental Protection Agency (https://www.epa.gov/tsca-screening-tools/epi-suitetm-estimation-program-interface).

### Care and use of *X. laevis* frogs

Oocytes in this study were surgically obtained from mature female *X. laevis* frogs. The care and use of frogs was carried out in accordance with the “Guidelines for Egg and Oocyte Harvesting in *X. laevis*, Revised 07/14/10” from the Animal Research Advisory Committee of the Office of Animal Care and Use at the National Institutes of Health and was approved by the Institutional Animal Care and Use Committee of the University of Miami. 0.1% 3-aminobenzoic acid ethyl ester was used to elicit anesthesia, as assessed by loss of nasal flare and swallow reflexes. Following surgical oocyte removal, the incision was sutured. A subcutaneous injection of Baytril (0.05 mL of a 2.27% solution) was administered as an antibiotic and a subcutaneous injection of Meloxicam (0.1 mL of a 0.015% solution) was administered to the dorsal lymph sack to serve as an analgesic, immediately following surgery. During recovery, frogs were kept in a humid environment before returning to the holding tank. Frogs were rested for at least 3 months between surgeries.

### Expression of Agam\Orco in *X. laevis* oocytes

A 2 hr incubation with collagenase B (Roche) was used to remove follicle cells from oocytes. Capped cRNA for Agam\Orco was synthesized using mMessage mMachine kits (Thermo Fisher Scientific). 25 ng of cRNA was injected into stage V–VI oocytes, followed by a 2–5 day incubation at 18 °C in Barth’s saline (in mM: 88 NaCl, 1 KCl, 2.4 NaHCO_3_, 0.3 CaNO_3_, 0.41 CaCl_2_, 0.82 MgSO_4_, 15 HEPES, pH 7.4, and 0.05 g/L tetracycline, 0.05 g/L ciprofloxacin, 0.1 g/L amikacin) prior to electrophysiological recordings.

### Electrophysiology and data capture

An automated parallel electrophysiology system (OpusExpress 6000 A, Molecular Devices) was used to record Orco agonist (OLC12) or odorant induced currents under two-electrode voltage clamp. Oocytes were perfused with ND96 (in mM: 96 NaCl, 2 KCl, 1 CaCl_2_, 1 MgCl_2_, 5 HEPES, pH 7.4). DMSO was used to prepare 1 M or 100 mM stock solutions of test compounds, which were then diluted in ND96 on the day of the experiment. Compounds were applied at a flow rate of 1.0 mL/min, with extensive washing in ND96 at 4.6 mL/min between applications. Micropipettes with resistances of 0.2–2.0 MΩ were filled with 3 M KCl. The holding potential was −70 mV. Current responses were filtered (4-pole, Bessel, low pass) at 20 Hz (−3 db) and sampled at 100 Hz. OpusXpress 1.1 software (Molecular Devices) was used to capture and store current responses.

### Experimental protocols and data analysis

To measure antagonist activity at Orco, Agam\Orco was activated by the Orco agonist OLC12^34^. Following two successive 60 sec applications of 30 μM OLC12, with 5 min washes between applications, each antagonist candidate was applied for 90 sec, immediately followed by a 60 sec co-application of antagonist candidate and OLC12. The 90 sec application of each compound alone (prior to co-application of the compound and OLC12) allowed us to ensure that antagonist candidate compounds were not causing non-specific effects on the oocytes that might confound the results. The current response to OLC12 in the presence of antagonist candidate was compared to the mean of the preceding two responses to OLC12 alone and presented as a percentage^34,35^. This value was then normalized to the value obtained when the assay was run in the absence of antagonist candidate (sham).

During development of the training panel (Fig. 2, Tables S1 and S2), all compounds not previously known to be Orco antagonists were tested at a concentration of 3 mM. Compounds eliciting ≤20 percent inhibition of the OLC12 response when applied at 3 mM were considered inactive. Concentration-inhibition curves were constructed for all compounds eliciting >20 percent inhibition at 3 mM. The same concentration and criteria were used during screening of predicted actives (Fig. 4D) and predicted inactives (Fig. 5C).

To measure the ability of Orco antagonists to inhibit odorant activation, oocytes expressing heteromeric ORs (Orco + ORX) were exposed to a 30 sec application of odorant followed by a 20 min wash. Oocytes were then exposed to a 90 sec application of Orco antagonist, immediately followed by a 30 sec co-application of Orco antagonist and odorant. The current response to odorant in the presence of Orco antagonist was compared to the preceding response to odorant alone and represented as a percentage. This value was then normalized to the value obtained when the assay was run in the absence of antagonist (sham).

Clampfit 9.1 software (Molecular Devices) was used to perform initial analysis of electrophysiological data. Prism 7 (Graphpad) was used for curve fitting. Concentration-inhibition data were fit to the equation: I = I_max_/(1 + (X/IC_50_)^n^) where I represents the current response at a given concentration of inhibitor, X; I_max_ is the maximal response in the absence of inhibitor; IC_50_ is the concentration of inhibitor present that allows a half maximal response from OLC12; n is the apparent Hill coefficient.

### Small molecule chemical structure standardization and management

Small molecule chemical structures along with screening results were curated and managed using ChemAxon JChem for Excel (version 16.6) and Instant JChem (version 16.6). Prior to performing computations, chemical structures were validated and standardized using ChemAxon Structure Checker (version 16.6) and Biovia Pipeline Pilot 2016. A total of 83 compounds tested in the Orco antagonist assay were processed. SDF files of 1280 small molecule structures from the Sigma-Aldrich Flavors and Fragrances catalog were obtained using the PerkinElmer Available Chemicals Exchange (ChemACX). These structures were then pre-processed as described above.

### Laplacian-corrected Naïve Bayesian classifiers

Laplacian modified Naïve Bayesian classifiers were used in combination with Extended Connectivity Fingerprints (ECFP4)^54^. This method is computationally efficient and has been shown in several studies to be robust and predictive^66,67^. However, these classifiers have not been previously applied to OR ligand discovery. A Naïve Bayesian classifier predicts active compounds based on the frequency of occurrence of chemical features in a training set of active and inactive compounds. A Laplacian correction accounts for the different sampling frequencies of the chemical features assuming that most features have no relation to activity; for example, Laplacian smoothing corrects the otherwise extreme effect on the predicted probability of a feature that was only observed once and happened to appear in the active class of the training set. Laplacian-corrected Bayesian classifiers were used as implemented in Biovia Pipeline Pilot 2016 (Biovia). ECFP4 circular fingerprints were chosen because they are unbiased and will produce a valid and meaningful topological description for any organic small molecules. We did not use structure keys (such as MACCS keys 166), because these are biased towards (human) drug-like compounds and are much less descriptive.

Two models were built, one (model A) using 58 antagonists (actives) and 25 inactives as the training set and another (model B) using only the most active antagonists with an IC_50_ of less than 500 μM (21 compounds) as actives and the same 25 inactives. Prior to model training, all chemical structures were standardized, then reviewed and curated using commercial catalogs and available databases, such as PubChem^68^. Extended connectivity fingerprints of radius 4 (ECFP4), AlogP, molecular weight, number of H-bond donors, number of H-bond acceptors, number of rotatable bonds, and the fractional polar surface area were used as molecular descriptors for the models. ROC scores were generated via the built-in model validation, which is based on leave-one-out cross validation. ROC scores were 0.89 for model A and 0.95 for model B. For further validation, the labels were randomized followed by model training, resulting in average ROC scores of 0.57 (stdD = 0.13) for model A and 0.58 (stdD = 0.09) for model B after 100 repetitions. In addition, for both datasets we performed k-fold stratified cross validations by splitting the actives and inactives datasets into equal k bins, each time training on all compounds except one bin and then testing using the left-out bin to compute an ROC score. ROC-scores of all k test sets were averaged. Because of the small number of compounds and because the number of actives and inactives are not multiples of k, for each k-fold cross validation a few left-over compounds were not used. Each k-fold cross validation experiment was repeated 1000 times, each time randomly reordering the training and test sets before splitting into k bins. This assures that the compounds are sufficiently sampled across training and test sets and that no compounds are left out altogether. The average k-fold ROC scores were then averaged across the 1000 runs. The ROC score results and exact numbers of actives and total compounds used for training are given in Table S3.

### Virtual screening of the Sigma-Aldrich Flavors and Fragrances catalog

Using the Laplacian-corrected Naïve Bayesian classifiers, predictions were made for 1280 valid chemical structures from the Sigma-Aldrich Flavors and Fragrances catalog. Normalized Bayesian scores, binary prediction (active/inactive) and the estimated probability of a sample being in the active class (EstPGood) were computed for each compound by both models. For a sample structure, EstPGood is derived from its position in a normal distribution of the training set scores obtained from leave-one-out cross validation calculations. Specifically, the normalized Bayesian score of the tested structure is inserted into a Gaussian curve expression using the mean and standard deviation calculated from the active training set’s Bayesian scores. This yields the estimated probability of the sample structure being in the “active” distribution. The process is repeated using the mean and standard deviation of the inactive training set class’ Bayesian scores, and EstPGood is finally calculated by dividing the “active” probability over the sum of itself and the “inactive” probability.

Novel actives were selected as follows: 138 molecules with predictions of EstPGood >0.9 in both models were selected. The compounds were clustered using the Pipeline Pilot Cluster Molecules component, which is based on a partitioning algorithm, using Tanimoto similarity and ECFP6 fingerprints. 5 molecules were defined as the average number per cluster resulting in 27 clusters. The 138 compounds selected based on EstPGood included 16 previously known actives that were part of the training set. After removing singletons from the clustering results, 39 additional compounds were manually selected such that at least one compound from each cluster was represented among the 138 predicted actives. In addition to structural diversity (by clustering) the selection criteria included molecular weight, the predicted probability EstPGood value, cluster size, and availability. Similarly, the most likely inactive compounds were identified by first filtering EstPGood <0.1 for both models and then clustering the 82 compounds into 16 clusters with an average of 5 members per cluster. The 82 most likely predicted inactives included 8 previously tested inactives that were part of the training set. 39 additional new compounds were selected to represent all clusters (except singletons) while also considering molecular weight, EstPGood value, cluster size, and availability. Maximum pairwise Tanimoto similarities for each of the 36 confirmed newly identified actives to all previously known actives (training compounds) were computed in Biovia Pipeline Pilot using Tanimoto similarity and ECFP4 fingerprints.

### *Drosophila melanogaster* electroantennogram (EAG) and single sensillum recording (SSR) assays

Adult flies (w1118 strain, 3–5 days old) were mounted in truncated pipette tips for detection of antennal responses^69,70^. EAG data were obtained with an IDAC-232 (Syntech), which was linked to a computer via the EAGpro data acquisition interface. SSR data were acquired with an IDAC 4 and AutoSpike (Syntech). Recording and indifferent electrodes were made of silver/silver chloride wires enclosed in glass capillary needles, which were pulled with a Micropipette Puller (P-97, Sutter Instruments Co.) and filled with 1 M potassium chloride in 1% polyvinylpyrrolidone. The indifferent electrode was inserted in the eye of an immobilized fly, while the EAG recording electrode was placed on the third segment of the antenna under a S2X12 Olympus microscope and by using a micromanipulator MP-12 (Syntech). For SSR, the recording electrode was inserted into a desired sensillum under a stage microscope (BX51WI, Olympus) and using a Piezo Manipulator (PM10, World Precision Instrument). The stimulus delivery system consisted of a Stimulus Controller CS-55 (Syntech), which delivered charcoal-filtered, humidified air at 650 mL/min through a stimulus tube (5 cm long; internal diameter, 8 mm). The outlet of the stimulus delivery tube was set 1 cm away from the preparations. Stimulus pulses (0.5 sec) were introduced through a hole in the stimulus delivery tube 2.5 cm away from outlet. A compensatory flow was applied through another hole in the tube (directly across from the stimulus pulse hole). The total flow (continuous plus compensatory or continuous plus stimulus) of 0.6 mL/s was measured at the center of the outlet of the stimulus delivery tube with an Anemometer Lite (Model 6006, Kanomax, Osaka, Japan). 10 µL of the odorants EA and 2 H (freshly diluted to 0.01%, 0.1% or 1% in mineral oil) or the Orco antagonist compounds BMP and LF (freshly diluted to 1% or 50% in mineral oil) were loaded onto filter papers (1 cm^2^). The filter papers containing odorants were placed inside of stimulus cartridges. Loading of odorant containing filter papers into the cartridges occurred in ambient air, which could contain trace amounts of odorants. Thus, stimulus pulses with ambient air (filter papers containing mineral oil alone) in the cartridges were used to define a baseline response. For EAG, filter papers containing BMP or LF (50% in mineral oil) were placed inside the main airflow tube 8 cm away from the antenna and odorants were delivered through a hole 2.5 cm away from the outlet. For SSR, BMP (1% in mineral oil) was delivered into the holes 2.5 cm away from the outlet, whereas the odorants were delivered upstream through a hole 4 cm away from the outlet. The different delivery arrangements may contribute to the difference in apparent BMP potency in the EAG and SSR experiments (Figs 6 and 7). Each filter paper was used 3–5 times before being replaced. CO_2_ was freshly collected into stimulus cartridge from the breath of the same subject (P.X.) and used only once. Spikes were manually counted 0.5 s before and 0.5 s after stimulus and the results presented as poststimulus number of spikes per 0.5 s (number of spikes after stimulus minus number of spikes prior to stimulus). Data were analyzed with One Way ANOVA using Prism 7 (GraphPad).

### *Drosophila melanogaster* larval chemotaxis assay

We have previously provided a detailed description of these methods^42^. Briefly, Canton-S (CS) flies were used as wild type (wt). The experimental chamber was a 100 × 15 mm polystyrene Petri dish (VWR) with 20 mL of 1.1% agarose coating the bottom. In this assay, 50 third-instar larvae were placed inside a “starting circle” at the center of the plate (the circle was drawn on the outside bottom of the plate). A line was also drawn that divided the plate in half. Small filter paper discs (d = 6 mm, Whatman Cellulose Filter Paper, # 1030 023) were placed on opposite sides of the plate (see Fig. 8A). Onto each filter paper disc was placed 10 μL of solution, containing either vehicle of dilution (mineral oil), odorant attractant (EA, diluted in mineral oil) or an Orco antagonist compound (diluted in mineral oil). The sides of the plate on which the compounds or vehicle were placed were alternated from experiment to experiment.

In order to test Orco antagonists for the ability to affect the chemotaxis assay, a large (d = 9 cm) filter paper (Whatman Filter Paper # D00161-S) was adhered with double-sided tape to the inside of the Petri dish lid. This allowed the larvae to be exposed to Orco antagonists in an airborne context. For light repulsion experiments, we used a Zeiss CL6000 LED light source at 76% intensity. Following the 5 min migratory period, plates were photographed, coded and then blind counted. Any larvae still within the starting circle or touching the center line were excluded from the analysis. A Response Index (RI) was calculated as RI = (S − C)/(S + C), where S is the number of larvae on the stimulus (EA) side and C is the number of larvae on the control (mineral oil) side. RI = 1 would indicate complete attraction, RI = −1 would indicate complete repulsion, and RI = 0 would indicate no preference. Statistical significance was assessed using a one-way analysis of the variance followed by Dunnett’s’s post-test.

## Supplementary information

Supplementary Info

## Data Availability

The datasets generated during the current study are available from the corresponding author on reasonable request.
