# Supplementary material for: Use of machine learning to identify novel, behaviorally active antagonists of the insect odorant receptor co-receptor (Orco) subunit
Source: Sci Rep. 2019 Mar 11;9:4055. doi: 10.1038/s41598-019-40640-4 (PMC6411751; doi:10.1038/s41598-019-40640-4)

Supplementary Information

for

Use of machine learning to identify novel, behaviorally active antagonists of the insect odorant receptor co-receptor (Orco) subunit

by

Devin Kepchia, Pingxi Xu, Raymond Terryn, Ana Castro, Stephan C. Schürer, Walter S. Leal and Charles W. Luetje

List of materials:

Table S1. Structures and potencies of compounds in Figure 2.

Table S2. Training set compounds found to be inactive.

Table S3. Characterization of Laplacian-corrected Naïve Bayesian classifiers (models A and B) by leave-one-out (l1o) and stratified k-fold cross-validation.

Table S4. Activity of identified Orco antagonists at odorant specificity subunits of *Drosophila melanogaster* (Dmel\Ors) and *Anopheles gambiae* (Agam\Ors).

Table S5. Compounds used in this study.

Figure S1. Structures of Orco agonists VUAA1 and OLC12

Figure S2. Application of Orco antagonists to sham (water) injected oocytes.

Figure S3. Effects of Orco antagonists on heteromeric ORs.

Figure S4. The extent of BMP inhibition of odorant responses in electroantennogram (EAG) recordings does not differ across different odorant concentrations.

Figure S5. The Orco antagonist BMP is a non-competitive inhibitor of an odorant agonist, but is a competitive inhibitor of an Orco agonist.

Figure S6. Results of the larval chemotaxis assay with Orco antagonists applied at 30 mM.

Table S1: Structures and potencies of compounds in Figure 2. IC_{50} values are presented as mean ± SEM (n = 3-8).

Compound	IC_{50} (µM)
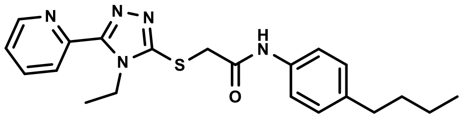 OLC15	10 ± 2
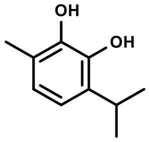 3-isopropyl-6-methyl-catechol (3I6MC)	41 ± 7
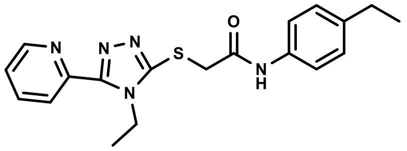 OLC2	45 ± 3
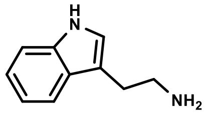 tryptamine	48 ± 10
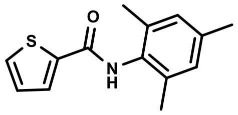 OX1L	58 ± 10
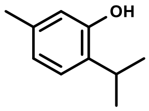 thymol	61 ± 10
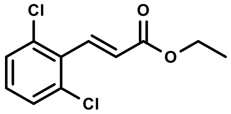 ethyl 2,6-dichlorocinamate	93 ± 11
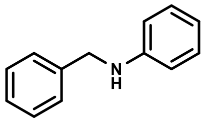 n-benzylaniline	114 ± 12
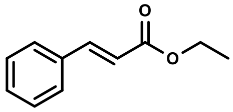 ethyl-trans-cinnamate	134 ± 16
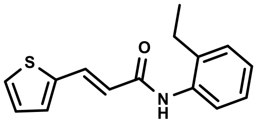 OX4	143 ± 34

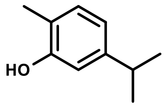 carvacrol	146 ± 21
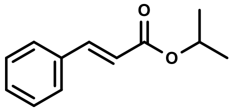 isopropyl cinnamate	153 ± 14
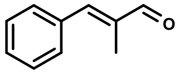 α-methyl cinnamaldehyde	187 ± 40
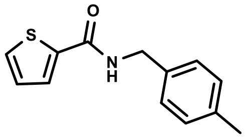 OX3a	202 ± 15
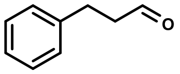 hydrocinnamaldehyde	220 ± 24
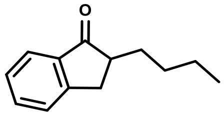 2-butyl-1-indanone	289 ± 28
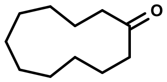 cycloundecanone	366 ± 28
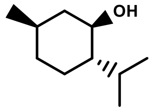 (-)menthol	433 ± 78
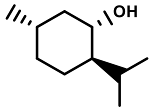 (+)menthol	443 ± 72
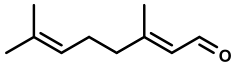 citral	452 ± 44
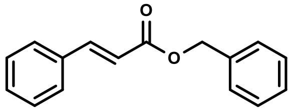 benzyl cinnamate	492 ± 64
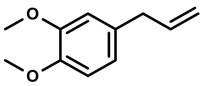 methyl eugenol	525 ± 40
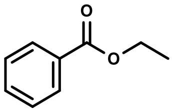 ethyl benzoate	569 ± 44
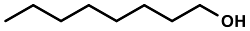	578 ± 16

1-octanol	
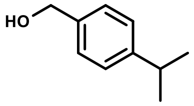 4-isopropylbenzyl alcohol	605 ± 116
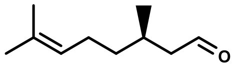 *(R)*-(+)-citronellal	651 ± 58
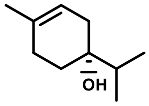 (−)-terpinen-4-ol	678 ± 78
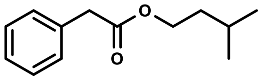 isoamyl phenylacetate	692 ± 66
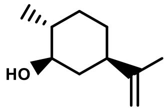 (-)-dihydrocarveol	818 ± 81
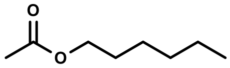 hexyl acetate	841 ± 121
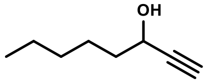 1-octyn-3-ol	870 ± 60
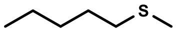 amyl methyl sulfide	915 ± 77
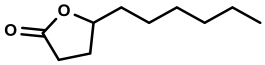 γ-decalactone	948 ± 153
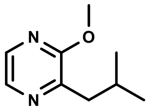 2-isobutyl-3-methoxypyrazine	1024 ± 83
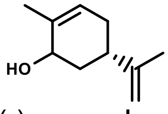 (-)-carveol	1027 ± 117
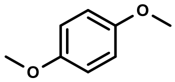 1,4-dimethoxybenzene	1101 ± 172
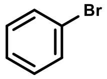 bromobenzene	1156 ± 143
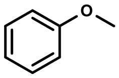 anisole	1169 ± 243
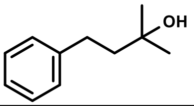	1240 ± 102

α,α-dimethylbenzenepropanol	
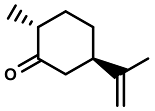<br>(+)-dihydrocarvone	1241 ± 219
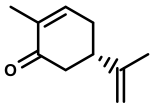<br>(*S*)-carvone	1277 ± 92
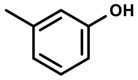<br>m-cresol	1285 ± 238
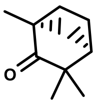<br>L-fenchone	1333 ± 105
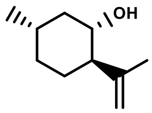<br>(+)-isopulegol	1370 ± 285
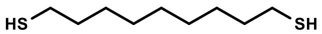<br>1,9-nonanedithiol	1596 ± 299
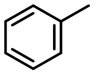<br>toluene	1602 ± 243
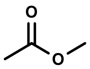<br>methyl acetate	1639 ± 179
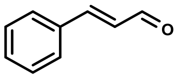<br>trans-cinnamaldehyde	1869 ± 94
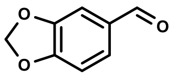<br>piperonal	2567 ± 244
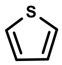<br>thiophene	2667 ± 147
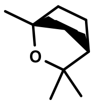<br>eucalyptol	2960 ± 255
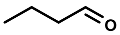<br>butyraldehyde	2998 ± 333
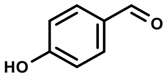<br>4-hydroxybenzaldehyde	3615 ± 449
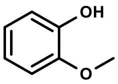<br>guaiacol	4034 ± 654

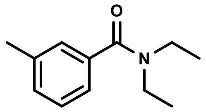 DEET	4478 ± 369
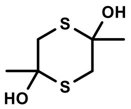 2,5-dimethyl-2,5-dihydroxy-1,4-dithiane	4862 ± 1269
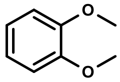 veratrole	5418 ± 845
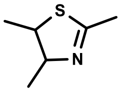 2,5-dihydro-2,4,5-trimethylthiazoline	12950 ± 2403

Table S2: Training set compounds found to be inactive (≤20% inhibition of Agam\Orco at 3 mM).

Compound	Structure
tryptophan	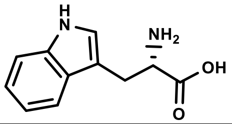
histidine	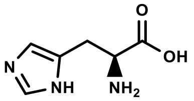
phenylalanine	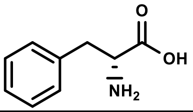
isophorone	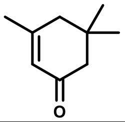
thiazole	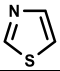
1-butanol	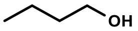
glucose	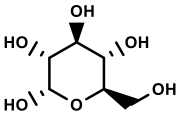
pyrazine	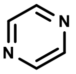
6-bromohexanoic acid	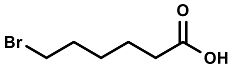
cyclopentanone	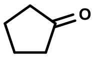
4-methylquinoline	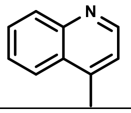
ethyl propionate	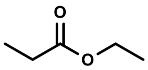
trimethylamine	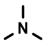
5-bromovaleric acid	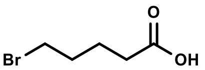
2,3-pentanedione	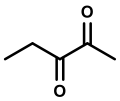
α-pinene	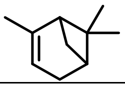
catechol	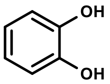
ethyl acetate	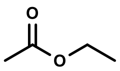
linoleic acid	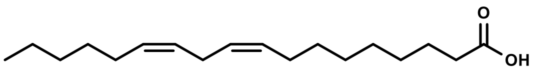

dimethyl succinate	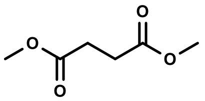
2-methoxy pyrazine	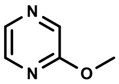
methyl isonicotinate	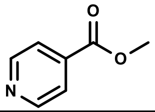
octanoic acid	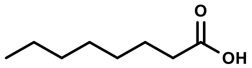
4-methyl-5-thiazoleethanol	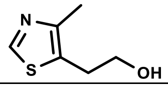
pyridine	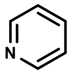

Table S3. Characterization of Laplacian-corrected Naïve Bayesian classifiers (models A and B) by leave-one-out (l1o) and stratified k-fold cross-validation. ROC AUC: area under the receiver operating characteristics curve; TP: true positives; FN: false negatives; FP: false positives; TN: true negatives. For l1o, the ROC score is a statistical estimate based on all predictions. Each of the k-fold stratified cross validation experiments (of k training/test runs) were repeated 1000 times.

**Model and description of experiment**	**CV fold**	**Num Actives**	**Num total**	**ROC AUC**	**TP / FN**	**FP / TN**
A (all actives and all inactives)	l1o	58	83	0.89	46/12	2/24
B (most potent actives and all inactives)	l1o	21	46	0.95	19/2	0/25
A shuffled (randomized labels; 100 repetitions)	l1o	~58	83	0.57 (stdD=0.13, n=100)	nd	nd
B shuffled (randomized labels; 100 repetitions)	l1o	~21	46	0.58 (stdD=0.09, n=100)	nd	nd
A (stratified cross validation; 1000 repetitions)	8	51	73	0.997 (stdD=0.004, n=1000)	nd	nd
B (stratified cross validation; 1000 repetitions)	8	19	41	1.0 (stdD=0.0, n=1000)	nd	nd
A (stratified cross validation; 1000 repetitions)	5	47	67	0.99 (stdD=0.0, n=1000)	nd	nd
B (stratified cross validation; 1000 repetitions)	5	17	37	1.0 (stdD=0.0, n=1000)	nd	nd
A (stratified cross validation; 1000 repetitions)	4	44	63	0.96 (stdD=0.01, n=1000)	nd	nd
B (stratified cross validation; 1000 repetitions)	4	16	35	1.0 (stdD=0.0, n=1000)	nd	nd
A (stratified cross validation; 1000 repetitions)	3	39	56	0.99 (stdD=0.0, n=1000)	nd	nd
B (stratified cross validation; 1000 repetitions)	3	14	31	0.97 (stdD=0.01, n=1000)	nd	nd
A (stratified cross validation; 1000 repetitions)	2	29	42	0.96 (stdD=0.01, n=1000)	nd	nd
B (stratified cross validation; 1000 repetitions)	2	11	24	0.99 (stdD=0.0, n=1000)	nd	nd

Table S4. Activity of identified Orco antagonists at odorant specificity subunits of *D. melanogaster* (Dmel\Ors) and *A. gambiae* (Agam\Ors). All active Orco antagonists in Figure 4D and Table 2 were compared to the Database of Odorant Responses (DoOR) [1] for Dmel\Or activity and to several publications [2-5] for Agam\Or activity. Whether the compound was part of the input training set or is a novel Orco antagonist is shown. The DoOR combines data from multiple studies into a scaled response (max = 1.0) for each odorant-OR pair. ORs displaying a scaled response > 0.1 are shown (if none > 0.1 then the Or with highest score is shown). Also, the OR displaying the greatest decrease in basal activity is shown.

Compound	CID	CAS	Input / Novel	Dmel\ORs (scaled responses from DoOR) [1]	Agam\ORs [2-5]
2-ethylphenol	6997	90-00-6	novel	Or10a (0.121), Or67b (0.236), Or69a (0.356)<br>Or47b (-0.168)	activation: Or2, Or9, Or10, Or11, Or14, Or18, Or20, Or38, Or41, Or50, Or57
4-hydroxy styrene	62453	2628-17-3	novel	Or71a (0.131)<br>Or19a (-0.028)	
4-isopropyl benzaldehyde	326	122-03-2	novel	Or19a (0.351), Or67a (0.339), Or85b (0.222)<br>Or22a (-0.011)	
4-phenyl-2-butanol	61302	2344-70-9	novel	Or71a (0.063)<br>Or19a (-0.007)	
citral	638011	5392-40-5	input	Or22a (0.140), Or67b (0.131), Or69a (0.483), Or82a (0.189), Or92a (0.325)<br>Or42b (-0.041)	
citronellal	7794	106-23-0	input	Or83c (0.092)	activation: Or30, Or31, Or48, Or50, Or75<br><br>inhibition of basal activity: Or5, Or12, Or15, Or25, Or45, Or73
ethyl cinnamate	637758	103-36-6	input	Or67a (0.143)<br>Or43a (-0.068)	
farnesal	5280598	19317-11-4	novel	Or83c (0.046)	
linalool	6549	78-70-6	novel	Or13a (0.164), Or19a (0.262), Or67a (0.158), Or69a (0.716), Or85b (0.163), Or98a (0.467),<br>Or42b (-0.042)	
p-cymene	7463	99-87-6	novel	Or71a (0.063)<br>Or65a (-0.028)	
trans-cinnamaldehyde	637511	14371-10-9	input	Or19a (0.112), Or22a (0.138)	

1. Münch, D., Galizia, C.G. DoOR 2.0 – Comprehensive mapping of Drosophila melanogaster odorant responses. *Scientific Reports* **6**, 21841 (2016).
2. Wang, G., Carey, A. F., Carlson, J. R. & Zwiebel, L. J. Molecular basis of odor coding in the malaria vector mosquito Anopheles gambiae. *Proc Natl Acad Sci U S A* **107**, 4418-4423 (2010).
3. Carey, A. F., Wang, G., Su, C. Y., Zwiebel, L. J. & Carlson, J. R. Odorant reception in the malaria mosquito Anopheles gambiae. *Nature* **464**, 66-71 (2010).
4. Xia, Y. *et al.* The molecular and cellular basis of olfactory-driven behavior in Anopheles gambiae larvae. *Proc Natl Acad Sci U S A* **105**, 6433-6438 (2008).
5. Lu, T. *et al.* Odor coding in the maxillary palp of the malaria vector mosquito *Anopheles gambiae*. *Curr Biol* **17**, 1533-1544 (2007)

Table S5. Compounds used in this study

Compound Name	Supplier	CID
(-) carveol	Sigma-Aldrich	7438
(-)-dihydrocarveol	Sigma-Aldrich	443163
(-)-menthol	Sigma-Aldrich	16666
(-)-terpinen-4-ol	Fluka	5325830
(+)-isopulegol	Sigma-Aldrich	1268090
(+)-dihydrocarvone	Sigma-Aldrich	24473
(+)-menthol	Sigma-Aldrich	165675
(+)-menthone	Sigma-Aldrich	443159
1-butanol	Sigma-Aldrich	263
1-octanol	Sigma-Aldrich	957
1-octyn-3-ol	Sigma-Aldrich	13166
1-pyrazinylethanone	Sigma-Aldrich	30914
1,4-dimethoxybenzene	Sigma-Aldrich	9016
1,9-nonanedithiol	Sigma-Aldrich	248488
10-undecylenic acid	Sigma-Aldrich	5634
(1*R*)-(-)-menthyl acetate	Sigma-Aldrich	12732529
2-acetonaphthone	Sigma-Aldrich	7122
2-butyl-1-indanone	Sigma-Aldrich	3404327
2-ethylphenol	Sigma-Aldrich	6997
2-isobutyl-3-methoxypyrazine	Sigma-Aldrich	32594
2-isopropylphenol	Sigma-Aldrich	6943
2-methoxypyrazine	Sigma-Aldrich	18467
2-methylpyrazine	Sigma-Aldrich	7976
2-phenylpropionaldehyde	Sigma-Aldrich	7146
2-tert-butyl-4-methylphenol	Sigma-Aldrich	17004
2-tert-butyl-6-isopropylphenol	Sigma-Aldrich	140960
2-tert-butyl-6-methylphenol (BMP)	Sigma-Aldrich	16678
2-tert-butylphenol	Sigma-Aldrich	6923
2,3-pentanedione	Sigma-Aldrich	11747
2,4-di-tert-butylphenol	Sigma-Aldrich	7311
2,4-diisopropylphenol	Sigma-Aldrich	18048
2,4-dimethyl-3-cyclohexene-1-carboxaldehyde	Sigma-Aldrich	93375
2,4-dimethylacetophenone	Sigma-Aldrich	6985
2,4-dimethylbenzaldehyde	Sigma-Aldrich	61814
2,4,6-trimethylphenol	Sigma-Aldrich	10698
2,5-dihydro-2,4,5-trimethylthiazoline	Contech	n/a
2,5-dimethyl-2,5-dihydroxy-1,4-dithiane	Sigma-Aldrich	62105
2,6-di-tert-butylphenol	Sigma-Aldrich	31405
2,6-diethylphenol	Sigma-Aldrich	70507
2,6-diisopropylphenol	Sigma-Aldrich	4943
3-isopropyl-6-methylcatechol (3I6MC)	Sigma-Aldrich	95873

3-isopropylphenol	Santa Cruz Biotech	12059
3-methoxy-p-cymene	Sigma-Aldrich	14104
3,5,5-trimethylhexanal	Sigma-Aldrich	21574
3,7-dimethyl-octadienenitrile	Sigma-Aldrich	1551246
4-ethylacetophenone	Sigma-Aldrich	13642
4-ethylbenzaldehyde	Sigma-Aldrich	20861
4-hydroxybenzaldehyde	Sigma-Aldrich	126
4-hydroxystyrene	Sigma-Aldrich	62453
4-isopropylbenzaldehyde	Sigma-Aldrich	326
4-isopropylbenzyl alcohol	Sigma-Aldrich	325
4-methyl-2-phenyl-pentenal	Sigma-Aldrich	5363896
4-methyl-5-thiazoleethanol	Sigma-Aldrich	1136
4-methylquinoline	Sigma-Aldrich	10285
4-phenyl-2-butanol	Sigma-Aldrich	61302
5-bromovaleric acid	Sigma-Aldrich	16368
6-bromohexanoic acid	Sigma-Aldrich	20210
6-tert-butyl-m-cresol	Sigma-Aldrich	6937
9-decenoic acid	Sigma-Aldrich	61743
acetoacetanilide	Sigma-Aldrich	7592
adipic acid	Sigma-Aldrich	196
allyl cinnamate	Sigma-Aldrich	641423
amyl methyl sulfide	Sigma-Aldrich	15620
anisole	Sigma-Aldrich	7519
benzaldehyde	Sigma-Aldrich	240
benzyl cinnamate	Sigma-Aldrich	5273469
benzyl isobutyrate	Sigma-Aldrich	7646
benzylacetone	Sigma-Aldrich	17355
beta-alanine	Sigma-Aldrich	239
bromobenzene	Sigma-Aldrich	7961
butyraldehyde	Sigma-Aldrich	261
butyric acid	Sigma-Aldrich	264
carvacrol	Sigma-Aldrich	10364
carvacrol methyl ether	Sigma-Aldrich	80790
catechol	Sigma-Aldrich	289
cinnamyl cinnamate	Sigma-Aldrich	1550890
citral	Sigma-Aldrich	638011
cyclopentanone	Sigma-Aldrich	8452
cycloundecanone	Sigma-Aldrich	13420
D-ribose	Sigma-Aldrich	10975657
DEET	Sigma-Aldrich	4284
dimethyl succinate	Sigma-Aldrich	7820
DL-alanine	Sigma-Aldrich	602
DL-malic acid	Sigma-Aldrich	525
enanthoic acid	Sigma-Aldrich	8094
ethyl methyl beta-phenylethyl carbinol	Sigma-Aldrich	61516

ethyl 2,6-dichlorocinamate	Sigma-Aldrich	6010430
ethyl acetate	Sigma-Aldrich	8857
ethyl benzoate	Sigma-Aldrich	7165
ethyl cinnamate	Sigma-Aldrich	637758
ethyl propionate	Sigma-Aldrich	7749
eucalyptol	Sigma-Aldrich	2758
farnesal	Sigma-Aldrich	5280598
glucose	Sigma-Aldrich	5793
glutamic acid	Sigma-Aldrich	33032
guaiacol	Sigma-Aldrich	460
hexanoic acid	Sigma-Aldrich	8892
hexyl acetate	Sigma-Aldrich	8908
histidine	Sigma-Aldrcih	6274
hydrocinnamaldehyde	Fluka	7707
isoamyl cinnamate	Sigma-Aldrich	5273467
isoamyl phenylacetate	Sigma-Aldrich	7600
isobutyl cinnamate	Sigma-Aldrich	778574
isophorone	Sigma-Aldrich	6544
isopropyl cinnamate	Sigma-Aldrich	5273464
L-aspartic acid	Sigma-Aldrich	5960
L-fenchone	Sigma-Aldrich	3034206
L-glutamine	Sigma-Aldrich	5961
L-leucine	Sigma-Aldrich	6106
L-lysine	Sigma-Aldrich	5962
L-methionine	Sigma-Aldrich	6137
L-ornithine monohydrochloride	Sigma-Aldrich	76654
L-valine	Sigma-Aldrich	6287
lauric acid	Sigma-Aldrich	3893
levulinic acid	Sigma-Aldrich	11579
linalool	Sigma-Aldrich	6549
linalyl formate (LF)	Sigma-Aldrich	61040
linoleic acid	Sigma-Aldrich	5280450
m-cresol	Sigma-Aldrich	342
m-tolualdehyde	Sigma-Aldrich	12105
methyl acetate	Sigma-Aldrich	6584
methyl eugenol	Sigma-Aldrich	7127
methyl isonicotinate	Sigma-Aldrich	227085
methyl nicotinate	Sigma-Aldrich	7151
methyl palmitate	Sigma-Aldrich	8181
methyl stearate	Sigma-Aldrich	8201
myristic acid	Sigma-Aldrich	11005
n-benzylaniline	Sigma-Aldrich	66028
n-decanoic acid	Sigma-Aldrich	2969
n-ethyl-p-methane-3-carboxamide	Sigma-Aldrich	24901712
n-hendecanoic acid	Sigma-Aldrich	8180

n-nonanoic acid	Sigma-Aldrich	8158
neohesperidin dihydrochalcone	Sigma-Aldrich	30231
octanoic acid	Sigma-Aldrich	379
OLC15 (N-(4-butylphenyl)-2-((4-Et-5-(2-pyridinyl)-4H-1,2,4-triazol-3-yl)thio)acetamide)	Sigma-Aldrich	n/a
OLC2	Sigma-Aldrich	1988610
oleic acid	Sigma-Aldrich	445639
OX1L (N-mesityl-2-thiophenecarboxamide)	Sigma-Aldrich	n/a
OX3a (N-(4-methylbenzyl)-2-thiophenecarboxamide)	Sigma-Aldrich	n/a
OX4	Sigma-Aldrich	2146484
p-cymene	Sigma-Aldrich	7463
p-tolualdehyde	Sigma-Aldrich	7725
palmitic acid	Sigma-Aldrich	985
pentadecanoic acid	Sigma-Aldrich	13849
phenethyl cinnamate	Sigma-Aldrich	5369459
phenylalanine	Sigma-Aldrich	6140
piperonal	Sigma-Aldrich	8438
pyrazine	Sigma-Aldrich	9261
pyrazineethanethiol	Sigma-Aldrich	61945
pyridine	Alfa Aesar	1049
(*R*)-(+)-citronellal	Sigma-Aldrich	75427
S-carvone	Sigma-Aldrich	16724
stearic acid	Sigma-Aldrich	5281
succinic acid	Sigma-Aldrich	1110
thiazole	Sigma-Aldrich	9256
thiophene	Sigma-Aldrich	8030
thymol	Sigma-Aldrich	6989
toluene	Sigma-Aldrich	1140
trans-cinnamaldehyde	Sigma-Aldrich	637511
tridecanoic acid	Sigma-Aldrich	12530
trimethylamine	Alfa Aesar	1146
tryptamine	Sigma-Aldrich	1150
tryptophan	Sigma-Aldrich	6305
tyrosine	Sigma-Aldrich	6057
valeric acid	Sigma-Aldrich	7991
veratrole	Sigma-Aldrich	7043
α-methyl-cinnamaldehyde	Sigma-Aldrich	7557
α-pinene	Sigma-Aldrich	6654
α,α-dimethylbenzenepropanol	Sigma-Aldrich	7632
γ-decalactone	Sigma-Aldrich	12813

Figure S1. Structures of Orco agonists VUAA1 and OLC12

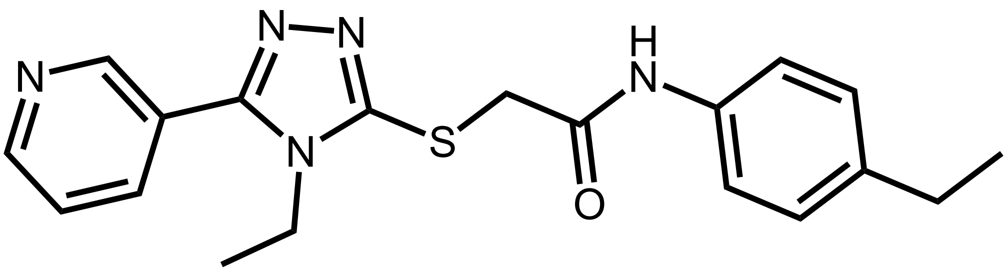

VUAA1

N-(4-ethylphenyl)-2-((4-ethyl-5-(3-pyridinyl)-4H-1,2,4-triazol-3-yl)thio)acetamide

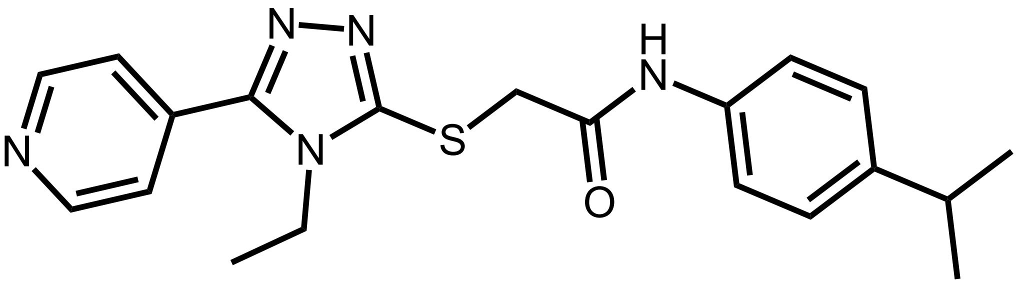

OLC12

2-((4-Ethyl-5-(4-pyridinyl)-4H-1,2,4-triazole-3-yl)sulfanyl)-N-(4-isopropylphenyl)acetamide

Figure S2. Application of Orco antagonists to sham (water) injected oocytes.
Upper trace, current recording from a sham (water) injected oocyte that was challenged with 60 sec applications of 100 µM and 1 mM 3-isopropyl-6-methyl-catechol (3I6MC). Representative of recordings from 4 oocytes. *Lower trace*, current recording from a sham (water) injected oocyte that was challenged with 60 sec applications of 3 mM 2-*tert*-butyl-6-methylphenol (BMP) and linalyl formate (LF). Representative of recordings from 4 oocytes.

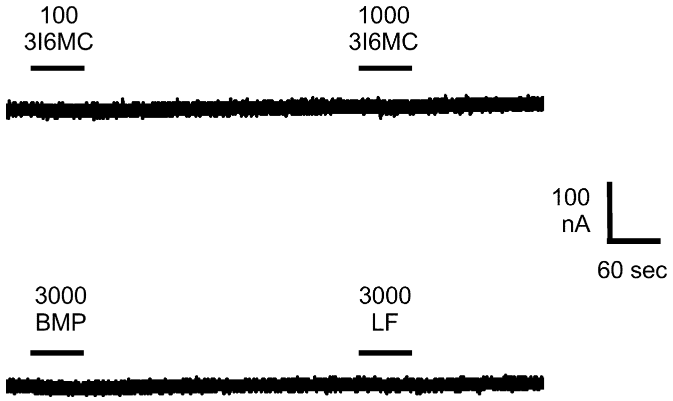

Figure S3. Effects of Orco antagonists on heteromeric ORs.
Here we show that three Orco antagonists can inhibit odorant activation of a variety of ORs. ORs are heteromeric complexes of odorant specificity subunits and Orco. The Orco antagonists tested were 3-isopropyl-6-methyl-catechol (3I6MC), a potent compound from the input training set, and two compounds identified in Figure 4 and Table 1: 2-*tert*-butyl-6-methylphenol (BMP) and linalyl formate (LF). These compounds were tested for antagonist activity against odorant activation of three ORs from *Anopheles gambiae* (Agam\Or28 + Agam\Orco, Agam\Or39 + Agam\Orco, Agam\Or65 + Agam\Orco), one OR from *Culex quinquefasciatus*, the southern house mosquito (Cqui\Or21 + Cqui\Orco) and one OR from *Drosophila melanogaster* (Dmel\Or35a + Dmel\Orco). Each OR was activated by its cognate odorant agonist applied at an approximate EC_{50} concentration. We also show that the Orco antagonists can antagonize OLC12 (Orco agonist) activation of heteromeric ORs. **A)** *Upper trace*, an oocyte expressing Agam\Or28 + Agam\Orco was challenged with a 30 sec application of 40 μM of the odorant acetophenone (ACE). After a 20 min wash period, 100 μM 3I6MC was applied for 90 sec before a second application of ACE and co-applied during the ACE application. *Lower trace*, an oocyte expressing Agam\Or28 + Agam\Orco was challenged with a 30 sec application of 40 μM ACE. After a 20 min wash period, 0.1% DMSO vehicle (sham) was applied for 90 sec before a second application of ACE and coapplied during the ACE application. **B)** Current responses of oocytes expressing Agam\Or28 + Agam\Orco activated by 40 μM acetophenone in the presence of 100 μM 3I6MC, 100 μM BMP or 100 μM LF were compared to the preceding response to acetophenone alone, normalized to the value obtained when the assay was run in the absence of antagonist (sham), and then presented as mean ± SEM (n = 3 - 6). **C)** Current responses of oocytes expressing Agam\Or39 + Agam\Orco activated by 10 μM of the odorant 6-methyl-5-hepten-2-one in the presence of 100 μM 3I6MC, 100 μM BMP or 100 μM LF were compared to the preceding response to 6-methyl-5-hepten-2-one alone, normalized to the value obtained when the assay was run in the absence of antagonist (sham), and then presented as mean ± SEM (n = 3). **D)** Current responses of oocytes expressing Agam\Or65 + Agam\Orco activated by 100 nM of the odorant eugenol in the presence of 100 μM 3I6MC, 100 μM BMP or 100 μM LF were compared to the preceding response to eugenol alone, normalized to the value obtained when the assay was run in the absence of antagonist (sham), and then presented as mean ± SEM (n = 4 - 6). **E)** Current responses of oocytes expressing Cqui\Or21 + Cqui\Orco activated by 30 nM of the odorant 3-methylindole in the presence of 100 μM 3I6MC, 100 μM BMP or 100 μM LF were compared to the preceding response to 3-methylindole alone, normalized to the value obtained when the assay was run in the absence of antagonist (sham), and then presented as mean ± SEM (n = 3 - 4). **F)** Current responses of oocytes expressing Cqui\Or21 + Cqui\Orco activated by 3 μM of Orco agonist OLC12 (approximate EC_{25}) in the presence of 100 μM 3I6MC, 100 μM BMP or 100 μM LF were compared to the average of two preceding responses to OLC12 alone, normalized to the value obtained when the assay was run in the absence of antagonist (sham), and then presented as mean ± SEM (n = 4). **G)** Current responses of oocytes expressing Dmel\Or35a + Dmel\Orco activated by 1 μM of the odorant hexanol in the presence of 100 μM 3I6MC, 100 μM BMP or 100 μM LF were compared to the preceding response to hexanol alone, normalized to the value obtained when the assay was run in the absence of antagonist (sham), and then presented as mean ± SEM (n = 4). **H)** Current responses of oocytes expressing Dmel\Or35a + Dmel\Orco activated by 10 μM of Orco agonist OLC12 (approximate EC_{25}) in the presence of 100 μM 3I6MC, 100 μM BMP, 300 μM BMP, 100 μM LF or 300 μM LF were compared to the average of two preceding responses to OLC12 alone, normalized to the value obtained when the assay was run in the absence of antagonist (sham), and then presented as mean ± SEM (n = 3 - 5). Statistical significance of the data in each panel was assessed by one-way analysis of variance followed by Dunnett's multiple comparison test using the pre-normalized values for each antagonist compared to the values obtained in the sham recordings (*, $p < 0.05$; **, $p < 0.01$; ***, $p < 0.001$).

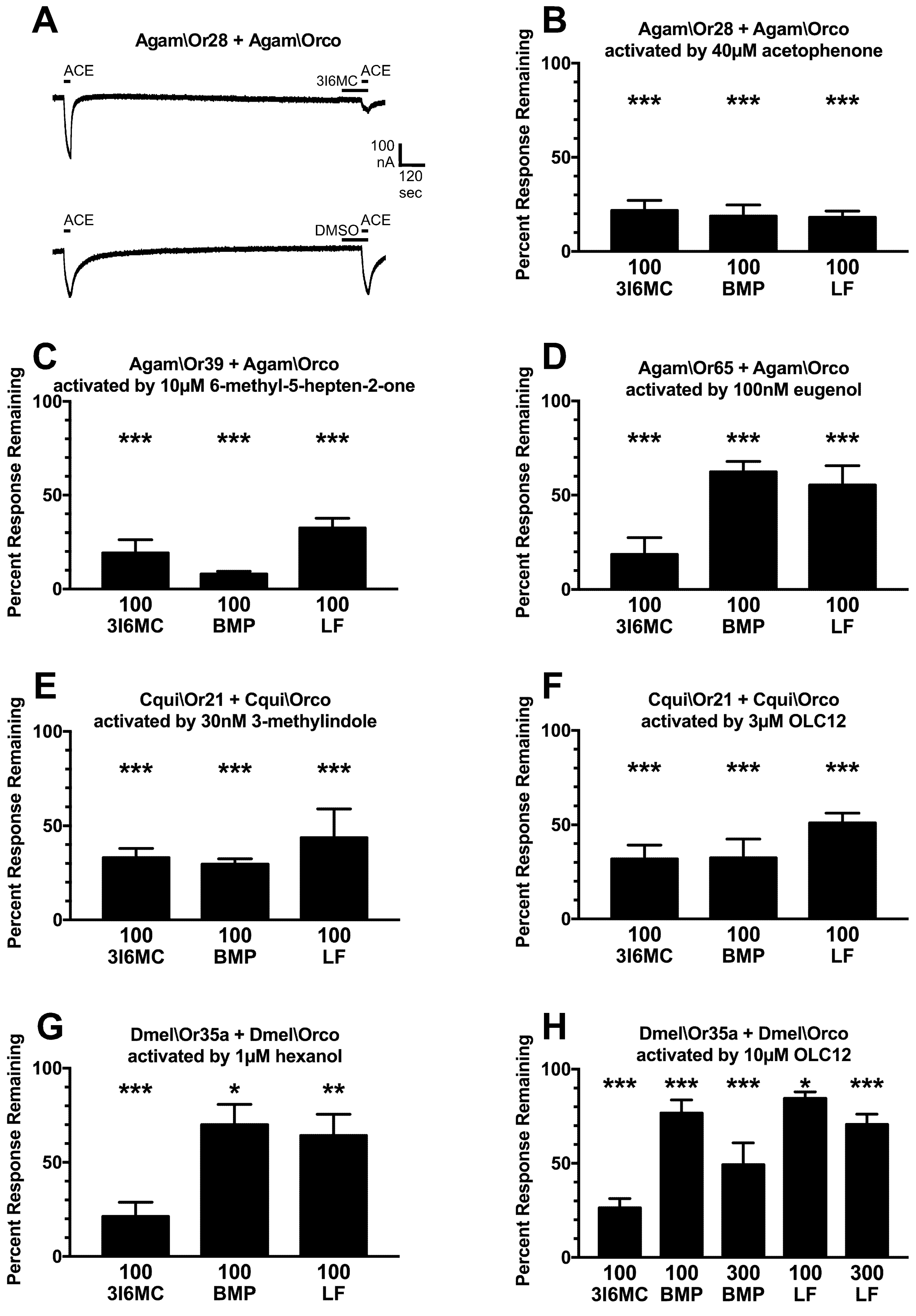
A
Agam\Or28 + Agam\Orco
ACE
3I6MC
ACE
100 nA
120 sec
ACE
DMSO
ACE
B
Agam\Or28 + Agam\Orco activated by 40μM acetophenone
Percent Response Remaining
100 3I6MC
100 BMP
100 LF
C
Agam\Or39 + Agam\Orco activated by 10μM 6-methyl-5-hepten-2-one
D
Agam\Or65 + Agam\Orco activated by 100nM eugenol
E
Cqui\Or21 + Cqui\Orco activated by 30nM 3-methylindole
F
Cqui\Or21 + Cqui\Orco activated by 3μM OLC12
G
Dmel\Or35a + Dmel\Orco activated by 1μM hexanol
H
Dmel\Or35a + Dmel\Orco activated by 10μM OLC12
100 3I6MC
100 BMP
300 BMP
100 LF
300 LF

Figure S4. The extent of BMP inhibition of odorant responses in electroantennogram (EAG) recordings does not differ across different odorant concentrations.
A) EAG responses (from Figure 6A) to stimulus pulses of 0.01%, 0.1% or 1% ethyl acetate (EA) in the presence of BMP are expressed here as a percentage of the preceding response to EA alone (mean ± SEM, n = 3). The extent of inhibition of the three different EA amounts did not differ (p=0.79, F-test).
B) EAG responses (from Figure 6A) to stimulus pulses of 0.01%, 0.1% or 1% 2-heptanone (2H) in the presence of BMP are expressed here as a percentage of the preceding response to 2H alone (mean ± SEM, n = 3). The extent of inhibition of the three different 2H amounts did not differ (p=0.57, F-test).

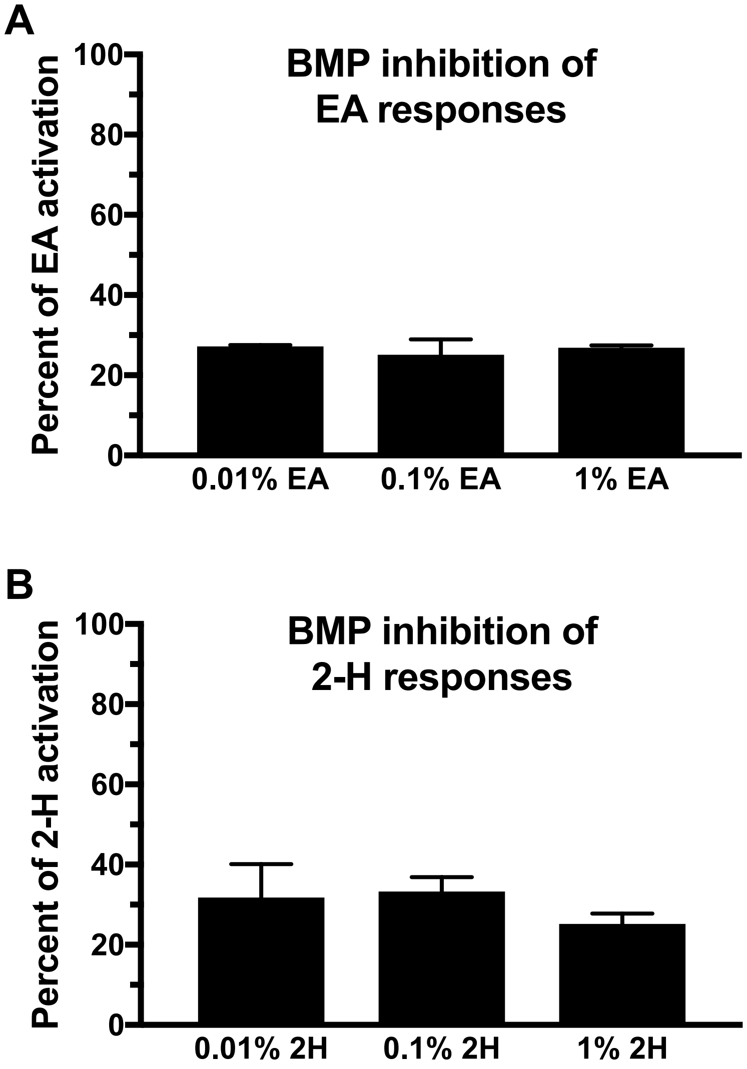

Figure S5. The Orco antagonist BMP is a non-competitive inhibitor of an odorant agonist, but is a competitive inhibitor of an Orco agonist.

A) Altering the concentration of the odorant acetophenone (ACE) fails to shift the BMP inhibition curve, indicating that BMP inhibition of odorant responses is non-competitive. Current responses of oocytes expressing Agam\Or28 + Agam\Orco activated by ACE (10 or 100 µM) in the presence of various concentrations of BMP were compared to the preceding response to ACE alone and then normalized to the values obtained when the assay was run in the absence of BMP (sham). IC_{50} values were derived from this concentration-inhibition data as described in Methods. The IC_{50} for BMP inhibition of Agam\Or28 + Agam\Orco activation by 10 µM ACE (35 ± 8 µM, n = 4-8) and the IC_{50} for BMP inhibition of Agam\Or28 + Agam\Orco activation by 100 µM ACE (53 ± 9 µM, n = 4-8) did not differ (p=0.166, F-test).

B) Altering the concentration of the Orco agonist OLC12 shifts the BMP inhibition curve, indicating that BMP inhibition of Orco agonist responses is competitive. Current responses of oocytes expressing Agam\Orco activated by the Orco agonist OLC12 (10 or 100 µM) in the presence of various concentrations of BMP were compared to the preceding responses to OLC12 alone and then normalized to the values obtained when the assay was run in the absence of BMP (sham). IC_{50} values were derived from this concentration-inhibition data as described in Methods. The IC_{50} for BMP inhibition of Agam\Orco activation by 10 µM OLC12 (12 ± 2 µM, n = 4-7) and the IC_{50} for BMP inhibition of Agam\Orco activation by 100 µM OLC12 (137 ± 38 µM, n = 3-9) were significantly different (p<0.0001, F-test).

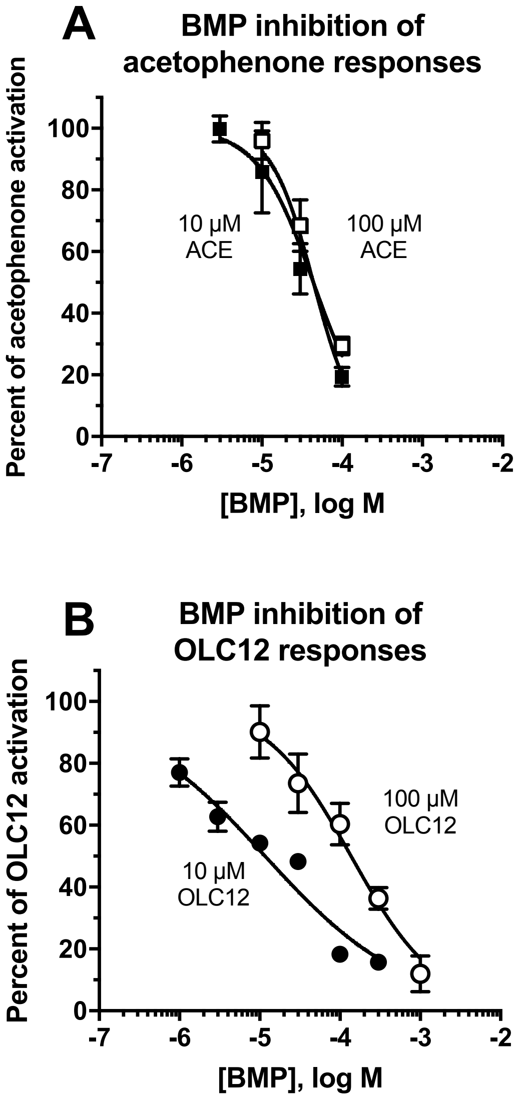

Figure S6. Results of the larval chemotaxis assay with Orco antagonists applied at 30 mM.
The assay was performed as described in Figure 8, except that BMP (2-*tert*-butyl-6-methylphenol) or LF (linalyl formate) were applied to the lid filter at a concentration of 30 mM. Data are presented as mean ± SEM (n = 4-8). Results were compared by one-way ANOVA and Dunnett's multiple comparison test for comparison to mineral oil (vehicle) in the lid filter (*, p<0.05).

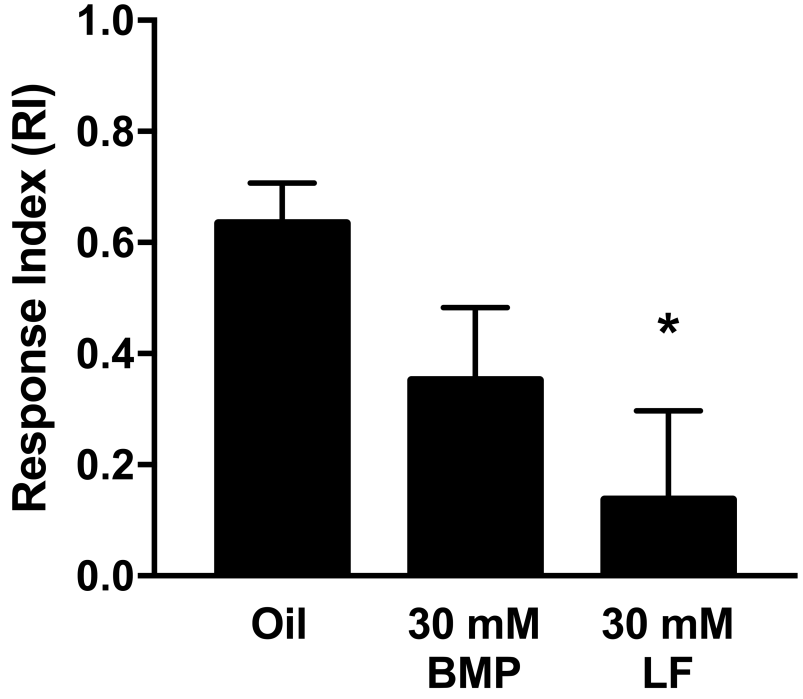

Supplement: Supplementary file 1 — Supplementary Info [file 41598_2019_40640_MOESM1_ESM.pdf]
